# Photobiomodulation in Dental Implant Surgery: A Systematic Review and Meta-Analysis of Randomized Controlled Trials

**DOI:** 10.3390/dj14090599

**Published:** 2026-09-16

**Authors:** Olívia Tosta de Macedo Vianna Mateus, Fernanda Rasch Czermainski, Tiago Henrique de Abreu Mateus, Albert Schiaveto de Souza

**Affiliations:** 1 Faculty of Medicine (FAMED), Federal University of Mato Grosso do Sul (UFMS), Campo Grande 79070-900, MS, Brazil; f.rasch@ufms.br; 2 Faculty of Engineering, Architecture and Urbanism and Geography (FAENG), Federal University of Mato Grosso do Sul (UFMS), Campo Grande 79070-900, MS, Brazil; tiago.mateus@ufms.br; 3 Institute of Biosciences (INBIO), Federal University of Mato Grosso do Sul (UFMS), Campo Grande 79070-900, MS, Brazil; albert.souza@ufms.br

**Keywords:** photobiomodulation, dental implants, osseointegration, systematic review, meta-analysis

## Abstract

**Background/Objectives**: Photobiomodulation (PBM) is proposed to enhance osseointegration, improve stability, reduce marginal bone loss, and alleviate pain in implant dentistry. Since clinical outcomes remain heterogeneous, this systematic review and meta-analysis evaluated PBM’s effects as an adjunct to implant therapy, focusing on stability, peri-implant bone outcomes, and pain. **Methods:** A comprehensive search of six databases and gray literature (June 2024–May 2025) followed PRISMA guidelines (PROSPERO: CRD420251041607). We included randomized controlled trials (RCTs) evaluating PBM against sham or no-PBM controls in adults. Risk of bias was assessed using RoB 2, and data were synthesized via random-effects meta-analyses with GRADE certainty assessments. **Results:** Thirty-two RCTs (829 patients, 1278 implants) were included. Postoperative pain was consistently reduced by PBM (MD = −0.27; 95% CI −0.35 to −0.19). For stability, Periotest favored PBM (MD = −0.55; 95% CI −0.84 to −0.27), indicating a transient early improvement. Similarly, resonance frequency analysis (ISQ) demonstrated a statistically significant improvement favoring the PBM group (MD = 1.26; 95% CI 0.61 to 1.91, *p* < 0.001), indicating a statistically significant increase in early implant stability. Marginal bone loss showed a statistically significant but clinically small reduction favoring PBM (MD = −0.09 mm; 95% CI −0.16 to −0.01). For peri-implant bone density, time-stratified models showed that PBM promoted a robust, significant initial benefit in 3D CBCT Hounsfield Units at baseline (MD = 114.07 HU, *p* = 0.01; overall MD = 86.02 HU, *p* = 0.14), while conventional 2D radiographic grayscale measurements captured a modest but statistically significant overall benefit (MD = 9.82, *p* = 0.03), showing a positive long-term trend up to 6 months (MD = 24.19, *p* = 0.04). **Conclusions:** PBM represents a promising supportive intervention in dental implant surgery, consistently reducing postoperative pain, enhancing early functional and structural stability, and promoting early bone mineralization. However, as the overall certainty of evidence according to the GRADE framework ranges from low to very low, these findings must be interpreted with caution due to substantial chronological and methodological heterogeneity across protocols.

## 1. Introduction

Photobiomodulation (PBM), previously referred to as low-level laser therapy, involves the application of red or near-infrared light to modulate cellular activity through interactions with mitochondrial chromophores, particularly cytochrome-c oxidase. This process enhances adenosine triphosphate (ATP) production, modulates reactive oxygen species, and promotes nitric oxide dissociation, activating signaling pathways associated with angiogenesis, osteogenic differentiation, and inflammatory regulation—mechanisms relevant to bone repair and peri-implant tissue homeostasis [1,2,3,4]. Recent advances have further clarified the role of mitochondrial signaling and cellular photoreception in mediating these effects [5,6].

The biological response to PBM is highly dependent on dosimetric parameters, including wavelength, irradiance, fluence, energy per point, exposure time, and number of sessions. These parameters follow a biphasic dose–response relationship, in which insufficient energy levels fail to elicit a therapeutic response, whereas excessive exposure may inhibit cellular activity [7,8,9,10]. Despite growing interest, substantial variability persists in how these parameters are applied and reported across studies, limiting reproducibility and hindering the establishment of standardized clinical protocols.

In implant dentistry, PBM has been proposed as an adjunctive strategy to influence osseointegration, improve early implant stability, reduce marginal bone loss, and alleviate postoperative pain. Preclinical evidence suggests that PBM may stimulate osteoblastic activity, collagen synthesis, and early mineralization, potentially affecting the bone–implant interface during healing [9,10,11,12,13]. However, translation of these findings into clinical practice remains uncertain. Clinical studies have reported heterogeneous outcomes, likely reflecting differences in PBM devices, irradiation protocols, surgical approaches, and outcome assessment methods [14,15].

Implant stability can be assessed using distinct methodologies, such as resonance frequency analysis (ISQ) and damping capacity (Periotest), which evaluate different biomechanical properties and may yield divergent results during healing [16,17,18]. Additionally, radiographic assessment of peri-implant bone changes, particularly bone density, varies considerably across studies, with differences in imaging modalities and measurement approaches further limiting comparability.

Previous systematic reviews have suggested potential benefits of PBM in implant dentistry; however, many were limited by small sample sizes, methodological heterogeneity, and inconsistent reporting of dosimetric parameters [14,15]. More recent meta-analyses have reported promising findings, particularly for early implant stability and postoperative pain, but also emphasize substantial variability across studies and the need for standardized protocols and improved methodological rigor [19,20]. Furthermore, the clinical relevance of some reported effects remains uncertain, highlighting the importance of distinguishing statistical significance from clinically meaningful outcomes.

Given the rapid expansion of PBM research and the persistence of methodological variability, an updated synthesis of randomized controlled trials is warranted. Therefore, this systematic review and meta-analysis aimed to evaluate the effects of PBM as an adjunct to dental implant therapy, focusing on implant stability, peri-implant bone outcomes, and postoperative pain, while incorporating risk-of-bias, subgroup, sensitivity, and GRADE assessments to provide a comprehensive and clinically relevant interpretation of the available evidence.

## 2. Materials and Methods

### 2.1. Study Design and Selection Process

This systematic review and meta-analysis followed the Preferred Reporting Items for Systematic Reviews and Meta-Analyses (PRISMA 2020 [21]) guidelines and adhered to the methodological standards outlined in the Cochrane Handbook for Systematic Reviews of Interventions [22]. Compliance with the PRISMA 2020 reporting standards was ensured, and the complete PRISMA checklist is provided in Supplementary Table S1. The protocol was prospectively registered in PROSPERO (CRD420251041607).

As this study is a systematic review and meta-analysis of the previously published literature, it did not involve direct interaction with human participants, animal subjects, or access to identifiable private patient data. Therefore, formal approval from an Institutional Review Board (IRB) or Ethics Committee was not required. Furthermore, all primary randomized controlled trials included in this review reported having obtained their respective local ethical approvals and informed consents prior to their conduction.

The review focused on RCTs [23], including parallel and split-mouth designs, evaluating PBM using low-level lasers or LEDs as an adjunct to dental implant placement in adults. The primary outcomes included implant stability—measured via resonance frequency analysis (ISQ) or Periotest—peri-implant bone density, marginal bone loss (MBL), and postoperative pain.

Study selection, data extraction, and risk-of-bias assessment were independently conducted by two reviewers, with disagreements resolved through consensus or consultation with a third reviewer.

### 2.2. Search Strategy

A comprehensive search strategy was developed to identify RCTs evaluating PBM in implant dentistry. Searches were performed across the following databases:

- MEDLINE (via PubMed and PMC);
- EMBASE;
- Scopus;
- ScienceDirect;
- Cochrane Library and CENTRAL.

No date restrictions were applied. To minimize publication bias, gray literature sources—including Google Scholar (first 200 results sorted by relevance) and ResearchGate—were also screened. Manual research was conducted in key journals related to PBM, photomedicine, and implant dentistry.

The search was conducted from June 2024 to May 2025. The reference lists of all included studies and relevant systematic reviews were screened for additional eligible articles. All the search strategies used in this review are fully reported in Supplementary Table S2.

To ensure transparency, reproducibility, and immediate accessibility, the complete electronic search strategy designed and applied for MEDLINE (via PubMed) is presented in Box 1.

Box 1 **PubMed/MEDLINE electronic search strategy.**

Wavelength/PBM Terms: (“photobiomodulation” OR “low level laser therapy” OR “low-level laser therapy” OR “LLLT” OR “laser therapy” OR “LED therapy” OR “light emitting diode” OR “near infrared” OR “red light therapy”)

AND

Implant/Osseointegration Terms: (“dental implants” OR “implant dentistry” OR “osseointegration” OR “oral implant” OR “implant stability”)

AND

Study Design Filters: (“randomized controlled trial” OR “randomised controlled trial” OR “controlled clinical trial” OR “RCT” OR “split mouth”)

NOT

Exclusion Filters: (animal OR mice OR rat OR canine OR “in vitro”)

Filters applied: Humans; Clinical Trial; Randomized Controlled Trial; English.

### 2.3. Eligibility Criteria (Table 1; Supplementary Tables S3–S6E)

Eligibility criteria were defined via the PICOS framework. Table 1 summarizes the predefined inclusion and exclusion criteria used to guide study selection, outlining the target population, PBM interventions, comparator groups, primary and secondary outcomes, and eligible study designs included in this systematic review and meta-analysis.

**Table 1:** Systematic review framework: PICOS elements and explicit eligibility criteria.

PICOS Element Selection Category	Description and Specifications
Part A: Core PICOS Framework	
Population (P)	Adults (≥18 years) undergoing dental implant placement in healed or immediate sites. Studies including medically controlled systemic conditions (e.g., controlled diabetes) were eligible if the condition did not inherently impair osseointegration. Pediatric populations were excluded.
Intervention (I)	PBM delivered via low-level lasers or LEDs, applied preoperatively, intraoperatively, and/or postoperatively. All wavelengths, dosimetry parameters, treatment geometries, and irradiation protocols were eligible.
Comparison (C)	Sham/placebo irradiation. No PBM (standard care). Studies comparing only different PBM parameters without a true control group were excluded.
Outcomes (O)	Primary outcomes: Implant stability (ISQ or Periotest). Peri-implant bone density. Marginal bone loss (MBL). Postoperative pain (VAS or NRS). Secondary outcomes (not required for inclusion): Peri-implant inflammation. Soft tissue healing. Postoperative complications.
Study Design (S)	Randomized controlled trials (parallel or split mouth). Non-randomized studies, observational designs, in vitro/animal studies, case reports/series, and reviews were excluded.
**Part B: Detailed Selection Criteria**	
Inclusion Criteria	RCTs evaluating PBM as an adjunctive therapy compared to a true inactive control (sham/placebo or untreated). Human subjects requiring dental implants. Peer-reviewed articles published in English with extractable quantitative outcome data.
Exclusion Criteria	Non-randomized trials, cohort studies, case series, animal/in vitro research, and literature reviews. Studies comparing only different active PBM parameters (e.g., Laser vs. LED) without an inactive control group. Cases involving extensive bone grafting, sinus lifts, or temporary orthodontic anchorage devices.

### 2.4. Unit of Analysis and Clustered Data

When studies reported multiple implants per patient, data were extracted at the patient level whenever possible. In cases where implant-level data were reported without adjustment for clustering, this was considered a potential unit-of-analysis error and addressed in sensitivity analyses.

Split-mouth studies were included and treated as paired data when sufficient information was available. When pairing information was missing, studies were included using conservative estimates, acknowledging potential overestimation of precision.

### 2.5. Data Extraction (Table 2)

Two reviewers independently performed study selection and data extraction using a piloted standardized form. To ensure consistency, the extracted data were cross-checked, and inter-reviewer agreement was assessed using Cohen’s kappa coefficient, demonstrating excellent agreement (κ = 0.89). Table 2 summarizes all study-level, participant-level, intervention-level, comparator, and outcome variables collected during data extraction.

**Table 2:** Domains and variables extracted from the included studies.

Domain	Variables Extracted
Study Characteristics	First author, publication year, country; Study design (parallel vs. split-mouth); Sample size and clinical setting.
Participant Characteristics	Number of patients and implants per group; Age, sex distribution, systemic conditions; Implant location (maxilla, mandible, or both); Timing of implant placement (immediate vs. delayed).
PBM Parameters	Device type (laser or LED); Wavelength (nm), power output (mW), irradiance (mW/cm^2^); Energy per point (J), fluence (J/cm^2^), spot size (cm^2^); Emission mode (continuous or pulsed); Number and anatomical location of irradiation points (buccal, lingual/palatal, crestal); Timing of PBM application and total number of sessions.
Comparator Characteristics	Description of sham/placebo irradiation or absence of PBM.
Outcome Measures	Extracted at all reported follow-up time points: Implant stability (ISQ or Periotest); Peri-implant bone density (radiographic or CBCT); Marginal bone loss (mm); Postoperative pain (VAS or NRS).

#### Handling of Missing Data

When SDs were not reported, they were calculated from SEs, CIs, IQRs, or *p* values via the Cochrane-recommended methods. Graphical data were extracted via WebPlotDigitizer (Ankit Rohatgi, Pacifica, CA, USA). When necessary, the study authors were contacted for clarification.

### 2.6. Risk of Bias

Two reviewers independently assessed the risk of bias via Cochrane’s risk of bias tool (RoB 2). The following domains were analyzed:

1. Bias arising from the randomization process;
2. Deviations from intended interventions;
3. Missing outcome data;
4. Outcome measurement bias;
5. Selective reporting bias.

Each domain was scored as low risk, some concerns, or high risk, with an overall judgment assigned accordingly. Discrepancies were resolved by consensus [24].

The full risk of bias profile, including domain-level judgments for each study, is provided in Supplementary Figure S1.

### 2.7. Statistical Analysis and Software

The meta-analyses were conducted using random-effects models (DerSimonian–Laird), considering the expected clinical heterogeneity in PBM parameters and follow-up intervals.

#### 2.7.1. Effect Measures

Continuous outcomes were synthesized using mean differences (MDs) with 95% confidence intervals (CIs). When necessary, scales were adjusted so that negative values consistently favored PBM. Periotest values were inverted as appropriate to maintain consistency in effect direction.

#### 2.7.2. Heterogeneity

Statistical heterogeneity was assessed using Cochran’s Q test and the *I*^2^ statistic, with the following thresholds: 0–25% (low), 26–50% (moderate), 51–75% (substantial), and >75% (considerable).

#### 2.7.3. Publication Bias

Publication bias was evaluated quantitatively using Egger’s regression test only for outcomes that included at least ten randomized controlled trials, in strict accordance with the Cochrane Handbook for Systematic Reviews of Interventions. For outcomes with fewer than ten studies, publication bias was assessed qualitatively through visual inspection of funnel plot asymmetry, as statistical tests for funnel plot asymmetry have extremely low power to distinguish chance from real asymmetry when the number of trials is limited [25,26].

#### 2.7.4. Software

All statistical analyses were performed using validated and widely adopted tools for systematic reviews and meta-analyses. Quantitative syntheses were conducted using Review Manager (RevMan, version 5.4; Cochrane, London, UK) and R software (version 4.5.2; R Foundation for Statistical Computing, Vienna, Austria), utilizing the ‘meta’ and ‘metafor’ packages for advanced statistical modeling, sensitivity analyses, and publication bias assessment.

#### 2.7.5. Risk of Bias and Certainty of Evidence

Risk of bias was assessed using the Cochrane Risk of Bias 2 (RoB 2) tool, and the certainty of evidence was evaluated using the GRADEpro GDT (GRADEpro Guideline Development Tool; McMaster University and Evidence Prime, Hamilton, ON, Canada) platform, enabling the generation of Summary of Findings (SoF) tables [26].

These tools ensured reproducible, transparent, and standardized workflows for data synthesis, visualization, and evidence grading.

### 2.8. Heterogeneity, Subgroup, and Sensitivity Analyses

Clinical heterogeneity across studies was evaluated based on differences in PBM parameters, implant site (maxilla vs. mandible), study design (parallel vs. split-mouth), and timing of postoperative assessments. Statistical heterogeneity was quantified using the *I*^2^ statistic and Cochran’s Q test, as described in Section 2.7.

Subgroup analyses were conducted to explore potential sources of heterogeneity when sufficient data were available (≥3 studies per subgroup). The following subgroups were predefined:

- Type of PBM device (laser vs. LED);
- Wavelength category (red: 620–699 nm vs. near-infrared: 700–980 nm);
- Energy dose (≤4 J per point vs. >4 J per point);
- Timing of outcome assessment (early vs. late);
- Measurement method for implant stability (ISQ vs. Periotest).

Differences between subgroups were assessed using χ^2^ tests for interaction and interpreted cautiously due to the limited number of studies and variability in dosimetric reporting.

Sensitivity analyses were performed to assess the robustness of pooled estimates, including:

- Leave-one-out analyses;
- Restriction to studies with low risk of bias according to RoB 2;
- Exclusion of studies with incomplete or unclear PBM parameters.

Variations in effect estimates were interpreted in the context of methodological and clinical differences across studies.

### 2.9. Certainty of Evidence (GRADE)

The certainty of evidence for each primary outcome—implant stability (ISQ and Periotest), peri-implant bone density, marginal bone loss (MBL), and postoperative pain—was assessed using the GRADE (Grading of Recommendations Assessment, Development and Evaluation) framework.

Two reviewers independently evaluated the certainty of evidence using the GRADEpro GDT platform, with disagreements resolved by consensus. The assessment considered five domains: risk of bias, inconsistency, indirectness, imprecision, and publication bias. Judgments were informed by RoB 2 assessments, variability in effect estimates and I^2^ values, relevance of study characteristics, confidence interval width, and potential publication bias assessed through funnel plots and Egger’s test when applicable.

According to GRADE guidance, randomized controlled trials were initially rated as high-certainty evidence and subsequently downgraded based on identified limitations. The final certainty levels were categorized as high, moderate, low, or very low.

Summary of Findings (SoF) tables were generated to present pooled estimates alongside certainty ratings, supporting transparent and clinically meaningful interpretation of the results.

## 3. Results

### 3.1. Study Selection (PRISMA-Figure 1)

A total of 87 records were identified through searches in databases and grey literature. After removing 22 duplicates, 65 records remained for screening by title and abstract.

Of these, 24 were excluded for not meeting the eligibility criteria (e.g., non-randomized studies, animal studies, or out-of-scope studies).

A total of 41 full-text articles were evaluated for eligibility. Of these, nine studies were excluded for the following reasons:

- Absence of an adequate control group (*n* = 2);
- Absence of PBM as an adjunct intervention (*n* = 1);
- Insufficient data for quantitative extraction (*n* = 3);
- Irrelevant outcomes (*n* = 3).

Thus, 32 randomized clinical trials were included in the qualitative and quantitative synthesis.

**Figure 1:**
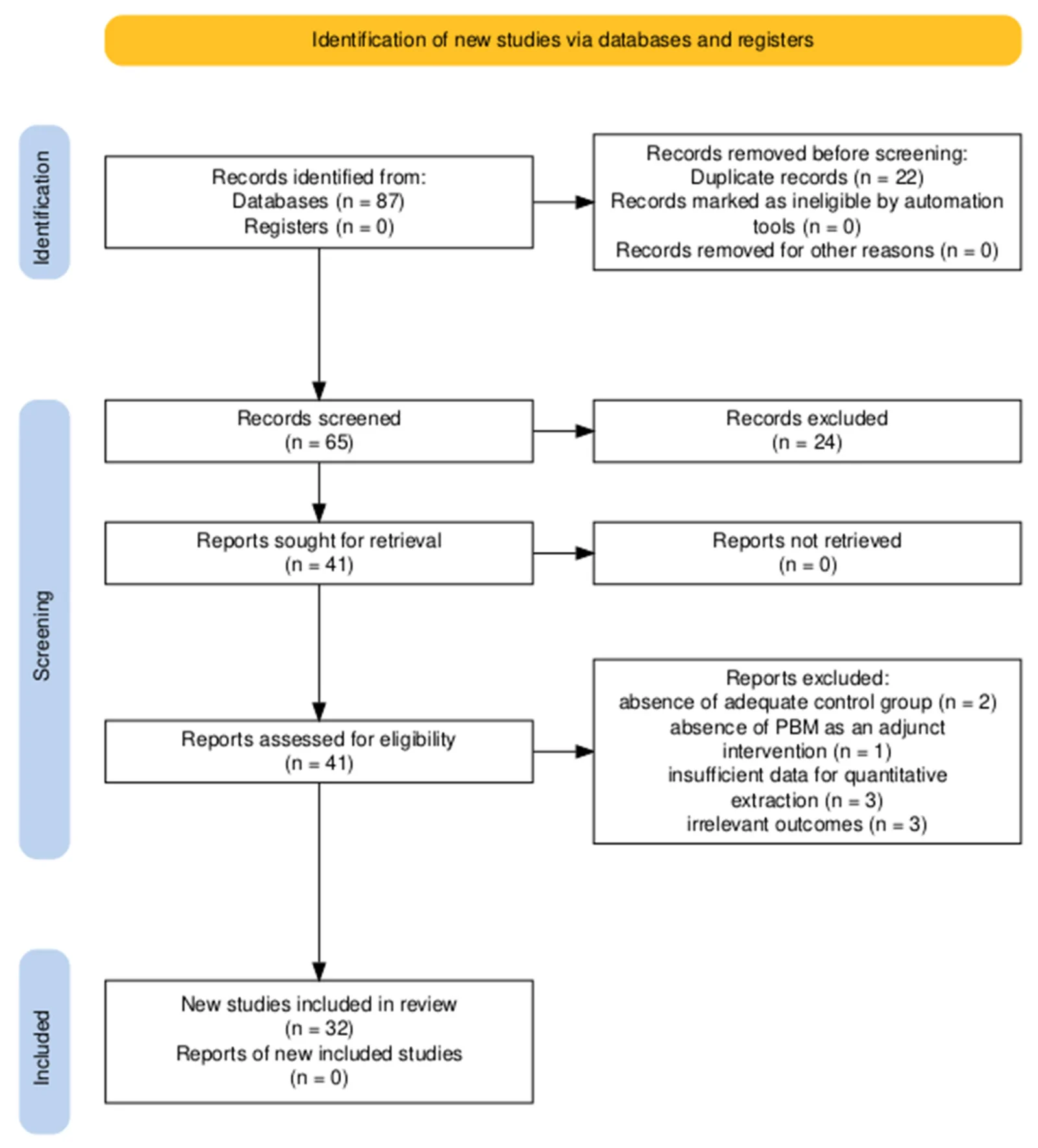
PRISMA flowchart of the criteria selected for the included articles.

The complete selection process is presented in the PRISMA 2020 flowchart (Figure 1) [21].

### 3.2. Characteristics of the Included Studies

The final sample consisted of 32 randomized controlled trials [27,28,29,30,31,32,33,34,35,36,37,38,39,40,41,42,43,44,45,46,47,48,49,50,51,52,53,54,55,56,57,58] identified through a comprehensive literature search conducted between June 2024 and May 2025, with no restriction on the starting publication date, conducted across diverse geographic regions, including Europe, Asia, South America, and the Middle East. The study designs included both parallel-group (*n* = 20) and split-mouth (*n* = 12) randomized trials. Of the 32 included trials, 30 provided quantitative data suitable for meta-analysis. Two trials (Basualdo Allende et al., 2024 [54]; and Caccianiga et al., 2020 [55]) were included strictly in the qualitative systematic synthesis but excluded from quantitative meta-analyses. This was because they did not report primary outcomes in a format convertible to Mean ± Standard Deviation (SD) (R54 reported outcomes strictly as percentage change scores, and R55 reported data as medians and ranges without standard deviations), and our attempts to contact the authors for raw data were unsuccessful. Sample sizes varied substantially, ranging from small exploratory trials with fewer than 20 participants to larger studies with over 60 enrolled subjects. Across all the studies, a total of 829 patients and 1278 implants were evaluated.

The participants were generally healthy adults, with mean ages ranging from the mid-20 s to the mid-60 s. Both maxillary and mandibular implant sites were represented, with several studies including mixed-arch placements. Implant systems varied, but most studies have employed titanium implants with standardized surgical protocols.

All included trials assessed the effect of PBM as an adjunct to implant placement, although PBM parameters differed widely across studies (see Section 3.3). The wavelengths ranged from 630 nm to 980 nm, the application modes included continuous and pulsed emission, and the number of postoperative irradiation sessions ranged from a single intraoperative exposure to multiple session protocols lasting up to two weeks.

The outcome assessment methods also varied. Implant stability was the most frequently reported outcome and was measured via resonance frequency analysis (ISQ) or Periotest devices. Bone-related outcomes, peri-implant bone density and MBL were assessed radiographically via periapical radiographs, panoramic radiographs, or CBCT. Postoperative pain was typically evaluated via VAS.

The follow-up durations ranged from immediate postoperative assessments to long-term monitoring for up to 12 months. Despite heterogeneity in study protocols, all trials reported sufficient data for inclusion in qualitative synthesis, and most provided extractable quantitative data for meta-analysis. A complete description of the demographic characteristics, implant features, PBM parameters, comparators, and outcome measures for each included study is provided in Supplementary Tables S3–S5.

### 3.3. PBM Parameters

Across the 32 included randomized controlled trials, PBM protocols exhibited substantial variability, reflecting the absence of standardized clinical guidelines for PBM application in implant dentistry. Differences were observed in device type, wavelength, power output, irradiation mode, energy delivered per point, total fluence, number of sessions, and anatomical application site (Supplementary Table S4).

#### 3.3.1. Device Type and Wavelengths

PBM was administered via low-level lasers or LEDs. The most frequently applied wavelengths fell within the red spectrum (630–699 nm) and near-infrared spectrum (780–980 nm). Laser devices are more common than LEDs are, and continuous-wave emission predominates over pulsed emission.

#### 3.3.2. Power Output, Energy Dose, and Fluence

The power outputs ranged from 20 mW to 200 mW, with energy doses varying considerably between studies, generally from 0.5 J to 8 J per point. The total delivered fluence frequently fell between 4 J/cm^2^ and 50 J/cm^2^, although several trials did not report spot size, power density, or irradiation area, limiting precise dosimetric comparisons.

#### 3.3.3. Application Protocols and Number of Sessions

The number of PBM sessions ranged from a single intraoperative application to multiple postoperative sessions, extending up to 14 days after implant placement. Combined intraoperative and postoperative protocols were used in a subset of studies, whereas others used only postoperative irradiation.

The application sites typically included:

- Buccal and lingual/palatal aspects of the implant site;
- Crestal areas;
- Periapical regions in some CBCT-guided protocols.

#### 3.3.4. Reporting Limitations

Several studies lacked complete dosimetric reporting—such as energy density, beam spot size, or irradiation distance—representing a recurring limitation and contributing to heterogeneity in effect estimates. These inconsistencies highlight the need for standardized PBM reporting guidelines in implant dentistry.

### 3.4. Meta-Analysis Outcomes

**Figure 2:**
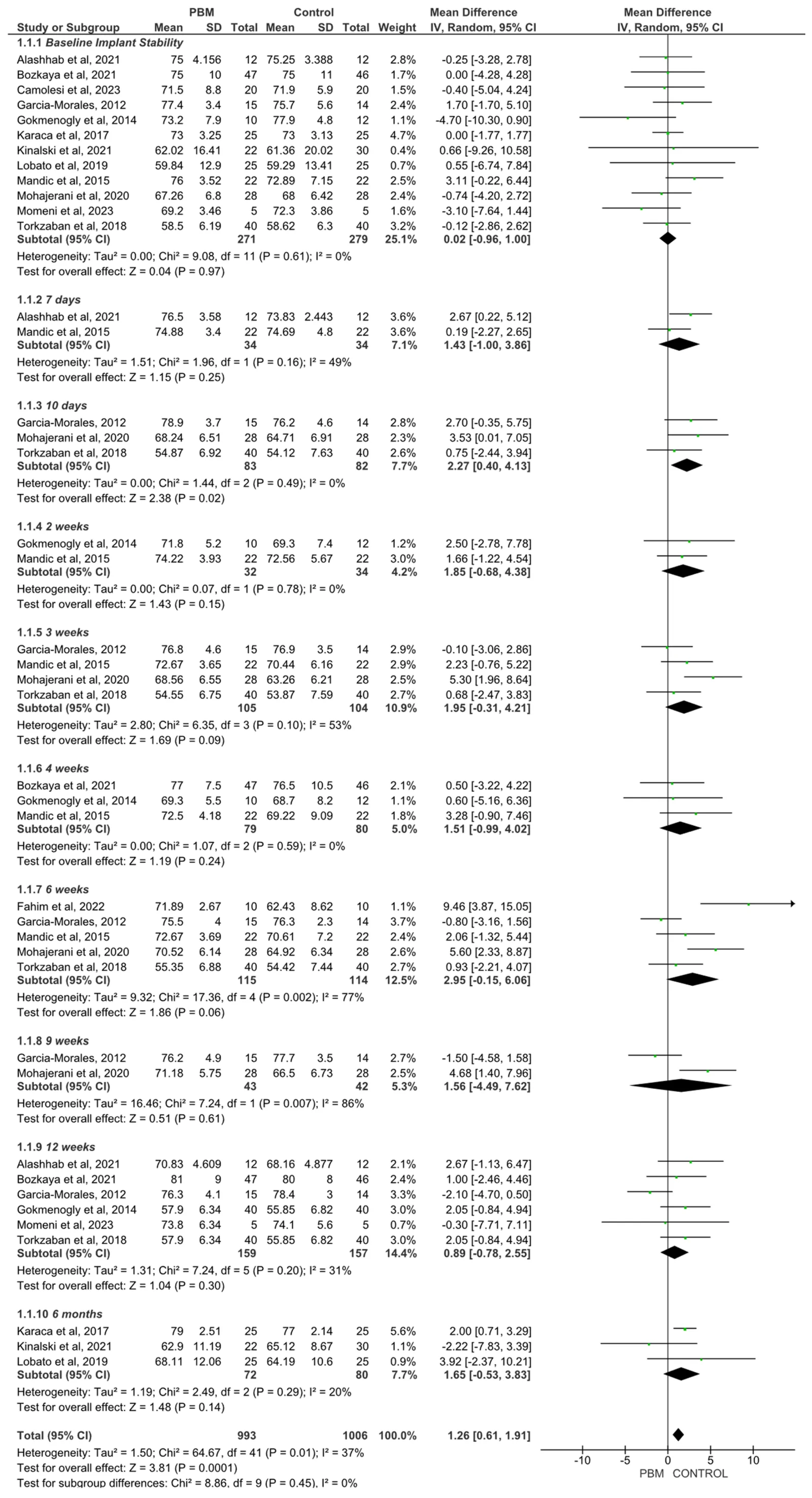
Forest plot of implant stability measured by resonance frequency analysis (ISQ) comparing PBM and control groups across randomized controlled trials [27,29,30,34,35,38,42,45,46,50,51,52,56]. Analyses were stratified by follow-up time, and random-effects models were applied due to expected clinical and methodological heterogeneity. The overall pooled estimate demonstrated a statistically significant difference favoring the PBM group (MD = 1.26; 95% CI 0.61 to 1.91; *p* < 0.001; *I*^2^ = 37%). Positive mean differences indicate higher ISQ values in the PBM group (favoring PBM), whereas negative values indicate higher ISQ values in the control group (favoring control). Green squares represent the effect estimates of individual studies, with horizontal lines indicating the corresponding 95% confidence intervals (CIs). Black diamonds represent the pooled effect estimates, with their widths indicating the corresponding 95% CIs. The vertical line at zero represents the line of no effect. Arrows indicate confidence intervals extending beyond the displayed scale of the forest plot.

**Figure 3:**
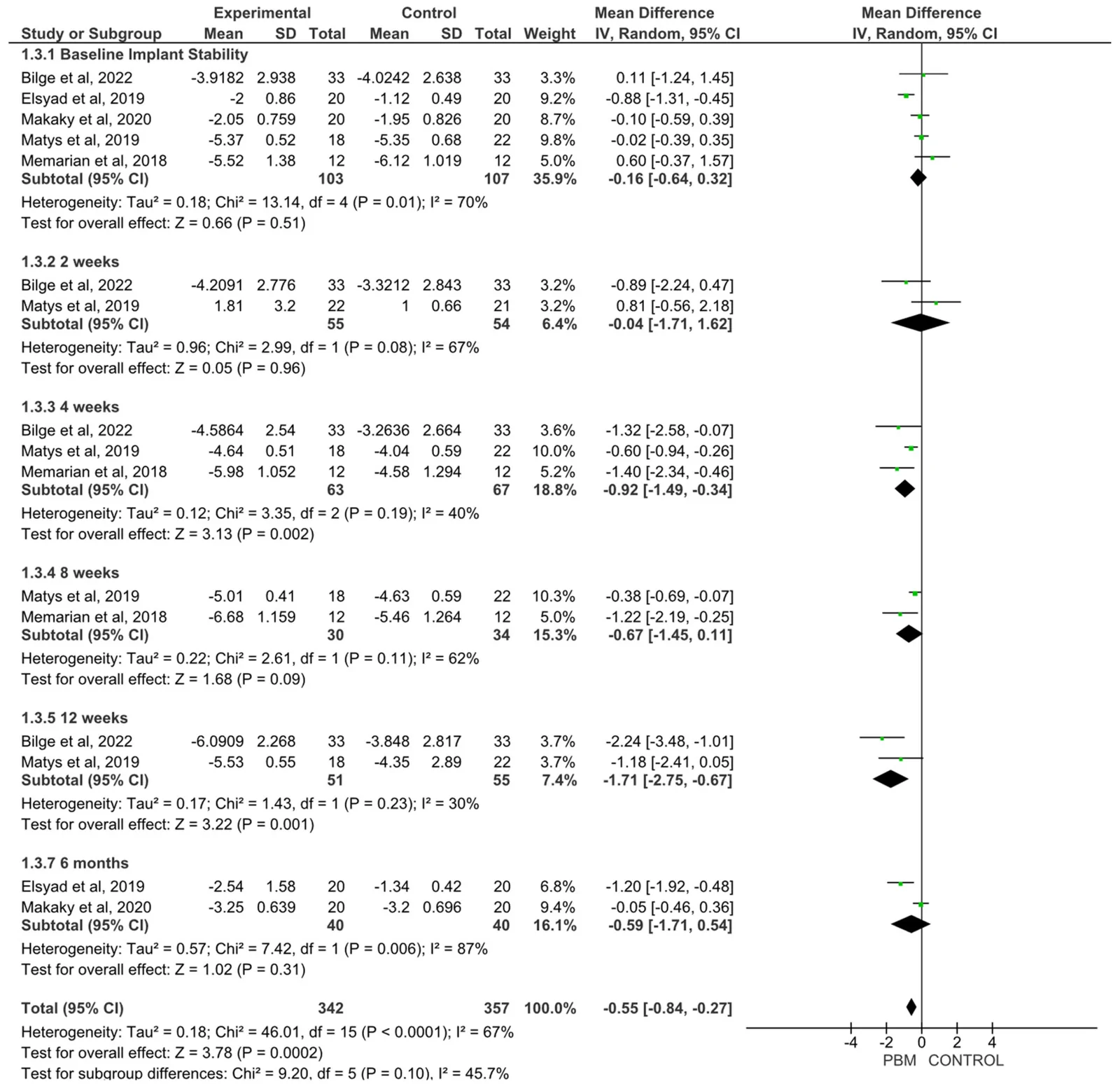
Forest plot of peri-implant stability measured by Periotest values comparing PBM and control groups across randomized controlled trials [32,33,37,47,49]. Analyses were stratified by follow-up time, and random-effects models were applied due to expected clinical and methodological heterogeneity. The overall pooled estimate demonstrated a statistically significant difference between groups favoring PBM (MD = −0.55; 95% CI −0.84 to −0.27; *p* = 0.0002; *I*^2^ = 67%). However, effects were not consistent across all time points. Negative mean differences indicate lower (i.e., better) Periotest values in the PBM group, whereas positive values favor the control group. Green squares represent the effect estimates of individual studies, with horizontal lines indicating the corresponding 95% confidence intervals (CIs). Black diamonds represent the pooled effect estimates, with their widths indicating the corresponding 95% CIs. The vertical line at zero represents the line of no effect. Arrows, where present, indicate confidence intervals extending beyond the displayed scale of the forest plot.

**Figure 4:**
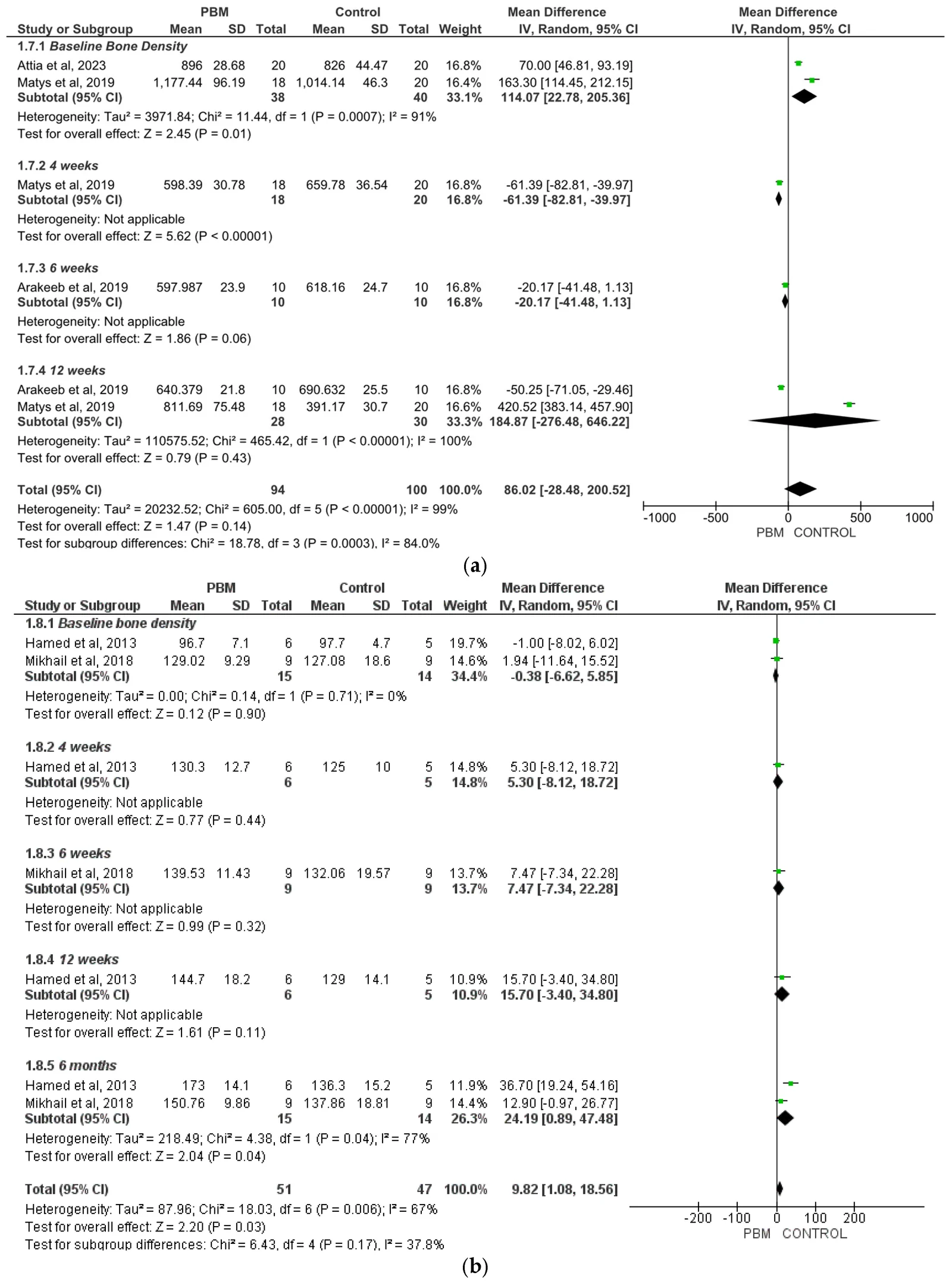
(**a**) Forest plot of peri-implant bone density assessed using three-dimensional (3D) CBCT-derived Hounsfield Units (HU), comparing the PBM and control groups stratified by follow-up intervals [33,40,41]. Positive mean differences indicate higher bone density values in the PBM group (favoring PBM), whereas negative values favor the control group. A random-effects model was applied to each follow-up subgroup. Extreme statistical heterogeneity (*I*^2^ = 99%) was observed, driven by chronological and methodological variability among the tomographic assessment protocols. (**b**) Forest plot of peri-implant bone density assessed using two-dimensional (2D) conventional radiographic arbitrary grayscale values (0–255), comparing the PBM and control groups stratified by follow-up intervals [28,31]. Positive mean differences indicate higher bone density values in the PBM group (favoring PBM), whereas negative values favor the control group. A random-effects model was applied, demonstrating a modest but statistically significant overall benefit favoring the PBM group across the pooled time points (MD = 9.82; 95% CI: 1.08 to 18.56; *p* = 0.03; *I*^2^= 67%). Green squares represent the effect estimates of individual studies, with horizontal lines indicating the corresponding 95% confidence intervals (CIs). Black diamonds represent the pooled effect estimates, with their widths indicating the corresponding 95% CIs. The vertical line at zero represents the line of no effect. Arrows, where present, indicate confidence intervals extending beyond the displayed scale of the forest plot.

**Figure 5:**
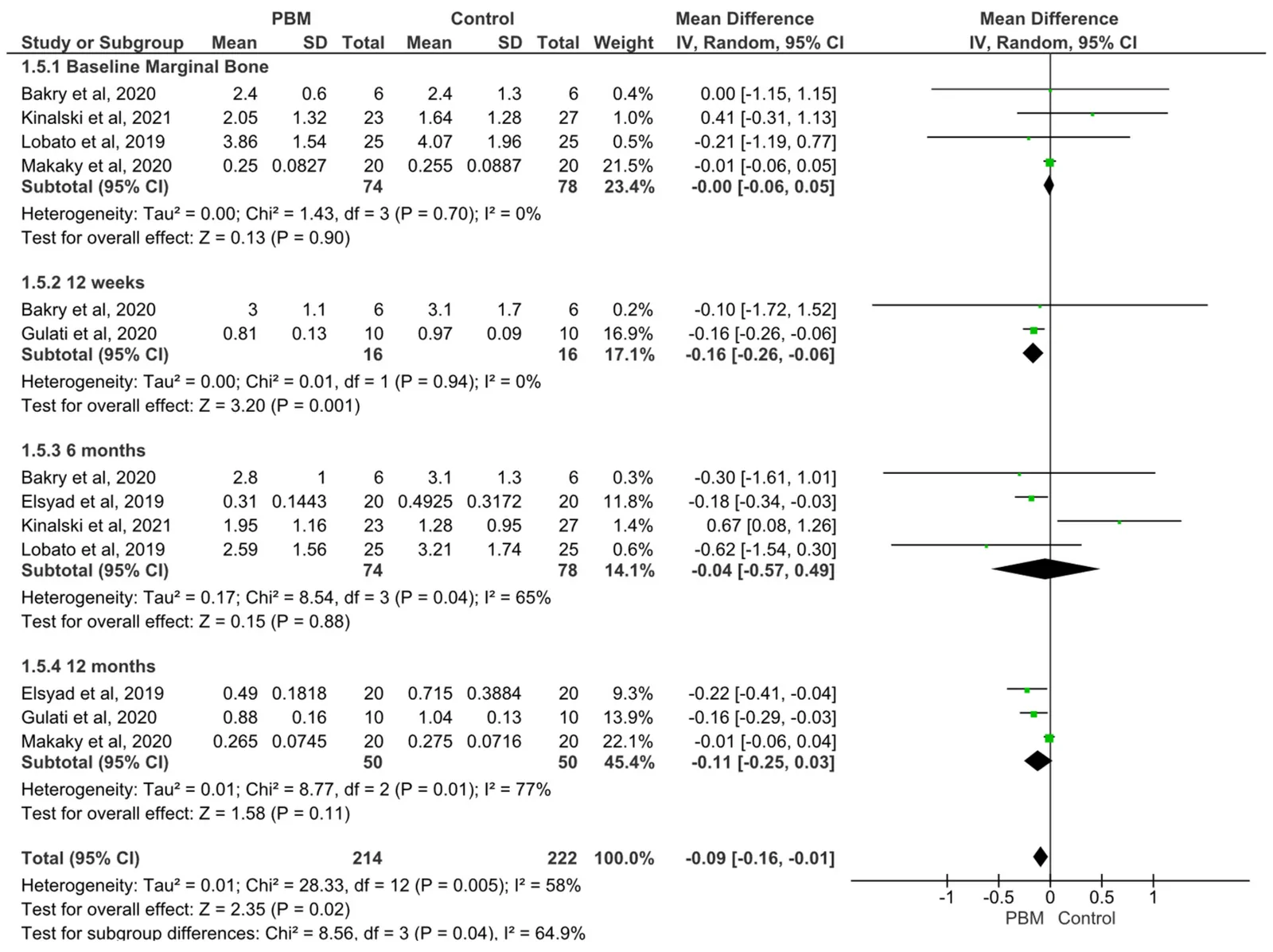
Forest plot of MBL comparing PBM and control groups across randomized controlled trials [32,35,39,46,47,53]. Analyses were stratified by follow-up time and conducted using random-effects models due to expected clinical and methodological heterogeneity. The overall pooled estimate demonstrated a statistically significant reduction in MBL favoring the PBM group (MD = −0.09 mm; 95% CI −0.16 to −0.01; *p* = 0.02; *I*^2^ = 58%). Subgroup analyses showed variable results across time points, with a significant effect observed at 12 weeks but not consistently at other follow-up periods. Negative mean differences indicate lower marginal bone loss (i.e., better outcomes) in the PBM group, whereas positive values favor the control group. Green squares represent the effect estimates of individual studies, with horizontal lines indicating the corresponding 95% confidence intervals (CIs). Black diamonds represent the pooled effect estimates, with their widths indicating the corresponding 95% CIs. The vertical line at zero represents the line of no effect. Arrows, where present, indicate confidence intervals extending beyond the displayed scale of the forest plot.

This section summarizes the quantitative findings from the five primary outcomes evaluated across the included randomized controlled trials. Forest plots for each outcome are presented in Figure 2, Figure 3, Figure 4 and Figure 5, and funnel plots assessing publication bias are provided in Supplementary Tables S5 and S6A–E, Figures S2 and S3.

#### 3.4.1. Implant Stability–ISQ (Figure 2; Supplementary Figures S2A and S3A)

A total of 13 studies reported resonance frequency analysis (RFA) outcomes expressed as ISQ values [27,29,30,34,35,38,42,45,46,50,51,52,56]. Implant stability was analyzed using a subgroup approach based on follow-up time to account for variability in measurement intervals across studies. Random-effects models were applied due to expected clinical and methodological heterogeneity (Figure 2).

At baseline, no significant difference between PBM and control groups was observed (MD = 0.02; 95% CI −0.96 to 1.00; *p* = 0.97; *I*^2^ = 0%), indicating comparable primary stability immediately after implant placement. In the early postoperative period, results were inconsistent. At 7 days, no significant effect was found (MD = 1.43; 95% CI −1.00 to 3.86; *p* = 0.25; I^2^ = 49%), whereas at 10 days, a statistically significant difference favoring the PBM group was observed (MD = 2.27; 95% CI 0.40 to 4.13; *p* = 0.02; *I*^2^ = 0%). At 2 weeks, the effect was not statistically significant (MD = 1.85; 95% CI −0.68 to 4.38; *p* = 0.15; *I*^2^ = 0%). In the intermediate follow-up period, findings remained variable. At 3 weeks, no significant difference was detected (MD = 1.95; 95% CI −0.31 to 4.21; *p* = 0.09; *I*^2^ = 53%), and at 4 weeks, results were also non-significant (MD = 1.51; 95% CI −0.99 to 4.02; *p* = 0.24; *I*^2^ = 0%). At 6 weeks, a borderline difference favoring the PBM group was observed (MD = 2.95; 95% CI −0.15 to 6.06; *p* = 0.06), accompanied by substantial heterogeneity (*I*^2^ = 77%). At 9 weeks, no significant difference was found (MD = 1.56; 95% CI −4.49 to 7.62; *p* = 0.61; *I*^2^ = 86%). At 12 weeks, which represented the most consistently reported time point across studies, no statistically significant difference between groups was observed (MD = 0.89; 95% CI −0.78 to 2.55; *p* = 0.30; *I*^2^ = 31%). At long-term follow-up (6 months), no significant difference between groups was detected (MD = 1.65; 95% CI −0.53 to 3.83; *p* = 0.14; *I*^2^ = 20%).

When considering all time points in a stratified analysis, the overall pooled estimate showed a statistically significant difference favoring the PBM group (MD = 1.26; 95% CI 0.61 to 1.91; *p* < 0.001), with moderate heterogeneity (*I*^2^ = 37%) (Figure 2). However, the test for subgroup differences was not statistically significant (*p* = 0.45), indicating that the effect did not differ consistently across time intervals. Overall, although some time points demonstrated statistically significant differences, these findings were not consistently observed across follow-up periods, and several subgroup estimates remained imprecise or heterogeneous.

Meta-regression analysis was performed to explore whether PBM energy dose influenced implant stability outcomes at 12 weeks. This analysis included six studies reporting ISQ measurements at this follow-up period. No statistically significant association was observed between dose and effect size (β = −0.076; 95% CI −0.295 to 0.143; *p* = 0.494). Residual heterogeneity remained high (*I*^2^ = 89.42%), and the proportion of heterogeneity explained by the model was negligible (R^2^ = 0%), indicating that PBM dose did not account for the variability observed across studies. However, given the limited number of studies included in the meta-regression, this analysis may have been underpowered to detect small or moderate associations. Therefore, the absence of a statistically significant relationship between PBM dose and implant stability should be interpreted with caution.

Nonetheless, a critical temporal observation must be highlighted: although the cumulative overall pooled estimate demonstrated a statistically significant benefit favoring the PBM group (MD = 1.26; 95% CI 0.61 to 1.91; *p* < 0.001), the majority of individual postoperative time points, when analyzed in isolation, did not achieve statistical significance. Consequently, these findings should be interpreted with caution, as the overall significance is largely driven by the cumulative sample size and statistical power across all pooled intervals, whereas the clinical effect of PBM at any single, isolated postoperative week remains modest.

#### 3.4.2. Implant Stability–Periotest (Figure 3; Supplementary Figures S2B and S3B)

Periotest values were reported in five studies [32,33,37,47,49]. Peri-implant stability assessed by Periotest values was analyzed using a time-stratified approach and random-effects models (Figure 3).

At baseline, no statistically significant difference between PBM and control groups was observed (MD = −0.16; 95% CI −0.64 to 0.32; *p* = 0.51; *I*^2^ = 70%). At 2 weeks, no significant effect was detected (MD = −0.04; 95% CI −1.71 to 1.62; *p* = 0.96; *I*^2^ = 67%). At 4 weeks, a statistically significant difference favoring PBM was observed (MD = −0.92; 95% CI −1.49 to −0.34; *p* = 0.002; *I*^2^ = 40%). At 8 weeks, the effect was not statistically significant (MD = −0.67; 95% CI −1.45 to 0.11; *p* = 0.09; *I*^2^ = 62%). At 12 weeks, a statistically significant difference favoring PBM was observed (MD = −1.71; 95% CI −2.75 to −0.67; *p* = 0.001; *I*^2^ = 30%). At 6 months, no significant difference between groups was found (MD = −0.59; 95% CI −1.71 to 0.54; *p* = 0.31; *I*^2^ = 87%). Overall, the pooled analysis demonstrated a statistically significant effect favoring PBM (MD = −0.55; 95% CI −0.84 to −0.27; *p* = 0.0002), with moderate heterogeneity (*I*^2^ = 67%) (Figure 3).

#### 3.4.3. Bone Density (Figure 4a,b; Supplementary Figures S2C,D and S3C)

Peri-implant bone density outcomes, assessed via radiographic or CBCT-based measurements, were reported in five randomized controlled trials. Due to the fundamental physical and mathematical incompatibility of pooling Hounsfield Units (3D CBCT absolute physical scale) and conventional grayscale values (2D arbitrary pixel scales from 0 to 255), outcomes were analyzed and interpreted strictly within their respective imaging modality subgroups, using time-stratified random-effects models.

For the three-dimensional (3D) CBCT (Hounsfield Units) subgroup (Figure 4a), the time-stratified pooled estimate demonstrated an overall mean difference of 86.02 HU (95% CI: −28.48 to 200.52; *p* = 0.14), with extreme heterogeneity (*I*^2^ = 99%). This high variability was driven by conflicting findings between follow-up intervals: while PBM promoted a statistically significant and robust bone density increase at baseline (MD = 114.07 HU; 95% CI: 22.78 to 205.36; *p* = 0.01), outcomes at subsequent early healing periods were highly variable, as seen at 4 weeks (MD = −61.39 HU; 95% CI: −82.81 to −39.97; *p* < 0.00001) and 6 weeks (MD = −20.17 HU; 95% CI: −41.48 to 1.13; *p* = 0.06) [33,40,41].

In contrast, the two-dimensional (2D) conventional radiography (grayscale) subgroup (Figure 4b) demonstrated a modest but statistically significant overall benefit favoring the PBM group across the pooled points of time (MD = 9.82; 95% CI: 1.08 to 18.56; *p* = 0.03; *I*^2^ = 67%). Subgroup analyses by follow-up period showed that while early density differences were non-significant at baseline (MD = −0.38; 95% CI: −6.62 to 5.85; *p* = 0.90), 4 weeks (MD = 5.30; 95% CI: −8.12 to 18.72; *p* = 0.44), 6 weeks (MD = 7.47; 95% CI: −7.34 to 22.28; *p* = 0.32), and 12 weeks (MD = 15.70; 95% CI: −3.40 to 34.80; *p* = 0.11), a statistically significant benefit favoring PBM was captured at the 6-month interval (MD = 24.19; 95% CI: 0.89 to 47.48; *p* = 0.04), indicating a positive long-term mineralizing trend [28,31].

#### 3.4.4. Marginal Bone Loss (MBL) (Figure 5; Supplementary Figures S2E and S3D)

Marginal bone loss was reported in six trials and analyzed using random-effects models (Figure 5). The pooled analysis demonstrated a small but statistically significant reduction in MBL in the PBM group compared with the control group (MD = −0.09 mm; 95% CI −0.16 to −0.01; *p* = 0.02), with moderate heterogeneity (*I*^2^ = 58%) [32,35,39,46,47,53].

Subgroup analyses showed variable results across follow-up periods. No significant differences were observed at baseline or at 6 months. At 12 weeks, a statistically significant reduction in MBL favoring PBM was observed (MD = −0.16; 95% CI −0.26 to −0.06; *p* = 0.001; *I*^2^ = 0%), whereas at 12 months, results were not statistically significant despite a tendency toward reduced MBL in the PBM group (MD = −0.11; 95% CI −0.25 to 0.03; *p* = 0.11; *I*^2^ = 77%) (Figure 5).

Despite statistical significance in the overall analysis, the magnitude of the effect was small and may not represent a clinically meaningful benefit. The clinical relevance of PBM in reducing marginal bone loss remains uncertain.

#### 3.4.5. Postoperative Pain (Figure 6; Supplementary Figures S2F and S3E)

**Figure 6:**
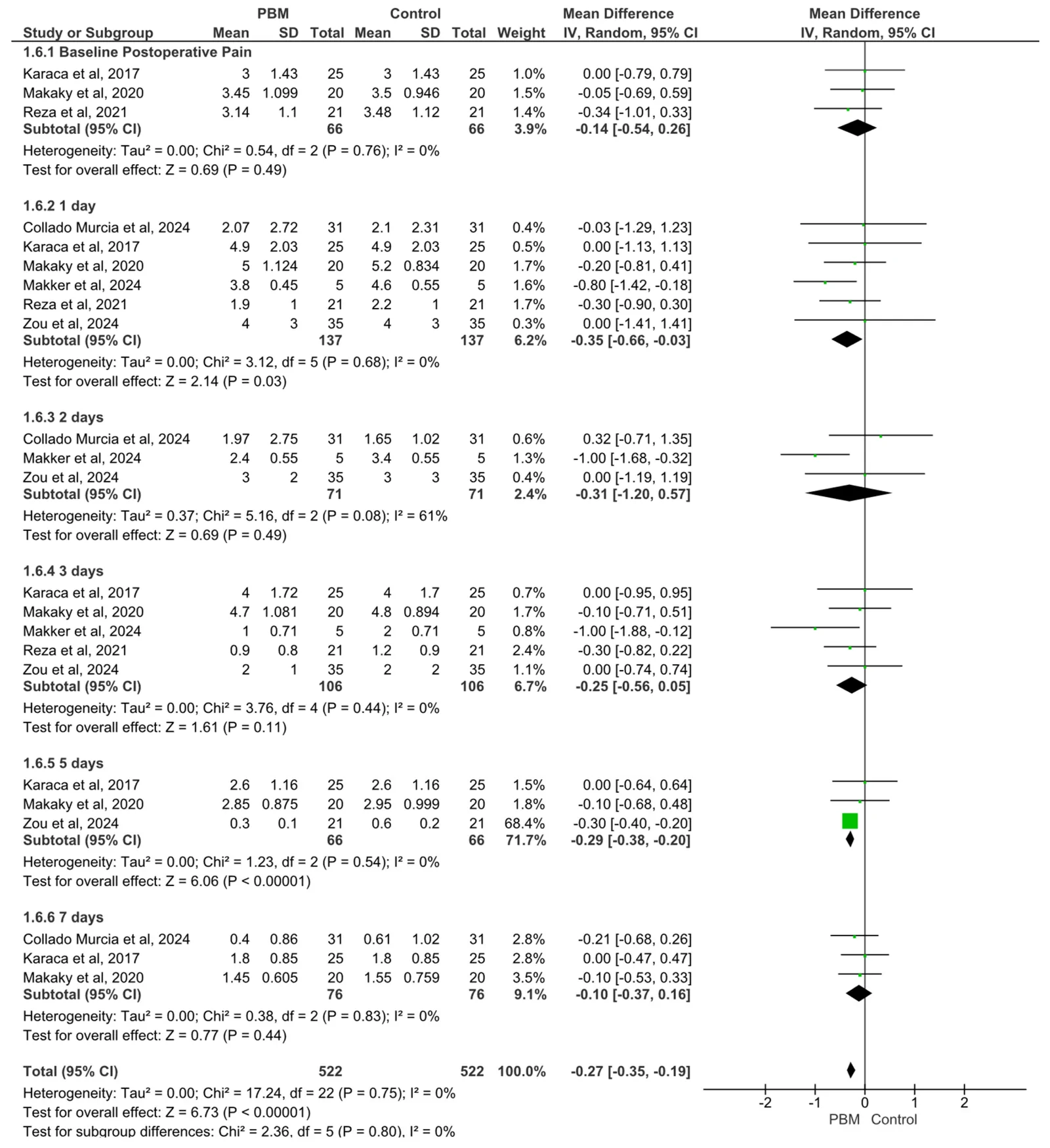
Forest plot of postoperative pain comparing PBM and control groups across randomized controlled trials [30,36,44,47,57,58]. Analyses were stratified by follow-up time and conducted using random-effects models. The overall pooled estimate demonstrated a statistically significant reduction in postoperative pain favoring the PBM group (MD = −0.27; 95% CI −0.35 to −0.19; *p* < 0.001; *I*^2^ = 0%). Subgroup analyses showed that this effect was more pronounced during the early postoperative period, although not all time points reached statistical significance. Negative mean differences indicate lower pain levels (i.e., better outcomes) in the PBM group, whereas positive values favor the control group. Green squares represent the effect estimates of individual studies, with horizontal lines indicating the corresponding 95% confidence intervals (CIs). Black diamonds represent the pooled effect estimates, with their widths indicating the corresponding 95% CIs. The vertical line at zero represents the line of no effect. Arrows, where present, indicate confidence intervals extending beyond the displayed scale of the forest plot.

Postoperative pain, primarily measured via VAS, was reported in 6 studies and analyzed using a time-stratified approach with random-effects models (Figure 6) [30,36,44,47,57,58]. At baseline (immediate postoperative period), no statistically significant difference between PBM and control groups was observed (MD = −0.14; 95% CI −0.54 to 0.26; *p* = 0.49; *I*^2^ = 0%). At 1 day, a statistically significant reduction in pain favoring PBM was observed (MD = −0.35; 95% CI −0.66 to −0.03; *p* = 0.03; *I*^2^ = 0%). At 2 days, no significant difference between groups was found (MD = −0.31; 95% CI −1.20 to 0.57; *p* = 0.49; *I*^2^ = 61%). At 3 days, no statistically significant difference was observed (MD = −0.25; 95% CI −0.56 to 0.05; *p* = 0.11; *I*^2^ = 0%). At 5 days, a significant reduction in pain favoring PBM was found (MD = −0.29; 95% CI −0.38 to −0.20; *p* < 0.001; *I*^2^ = 0%). At 7 days, no significant difference was observed (MD = −0.10; 95% CI −0.37 to 0.16; *p* = 0.45; *I*^2^ = 0%). Overall, the pooled analysis demonstrated a statistically significant reduction in postoperative pain favoring the PBM group (MD = −0.27; 95% CI −0.35 to −0.19; *p* < 0.001), with no observed heterogeneity (*I*^2^ = 0%). These findings suggest a consistent effect of PBM in reducing postoperative pain, particularly in the early postoperative period.

### 3.5. Risk of Bias Assessment (Supplementary Figure S1)

**Figure 7:**
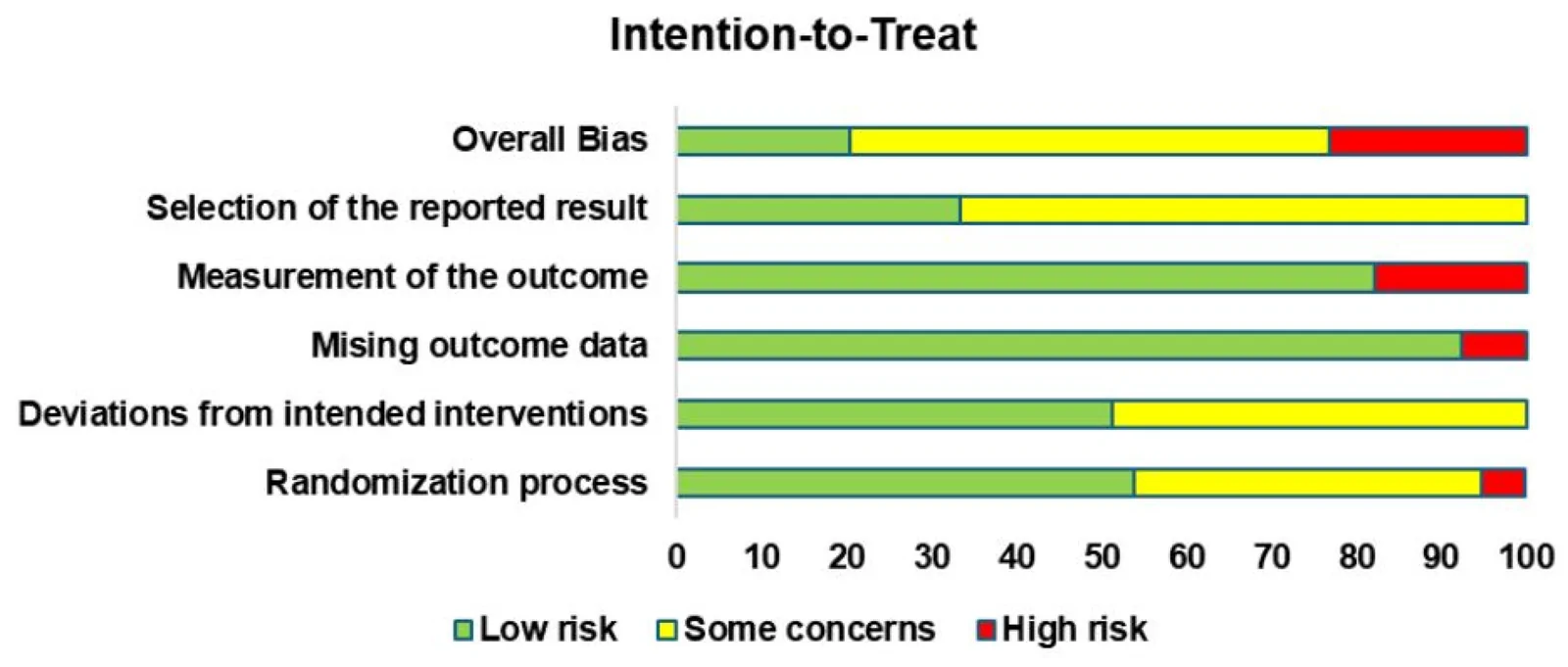
Risk of bias summary: review authors’ judgements about each risk of bias item presented as percentages across all included studies.

Risk of bias was assessed via the Cochrane Risk of Bias 2 (RoB 2) tool by two independent reviewers across five domains: randomization process, deviations from intended interventions, missing outcome data, outcome measurement, and selection of the reported result. The overall cumulative risk of bias assessment across all evaluated domains is visually summarized in Figure 7. Detailed study-by-study judgments are presented in Supplementary Figure S1.

Overall, most trials were judged as having a low risk of bias or some concerns, with a limited number classified as having a high risk for specific outcomes. Bias arising from the randomization process was generally low; however, unclear reporting of allocation concealment led to some concerns in several studies.

Most trials showed a low risk for deviations from intended interventions, particularly those using sham irradiation, although incomplete blinding in no-treatment control designs contributed to some concerns. Missing outcome data was generally minimal and balanced between groups, resulting in a low risk of attrition bias in most studies.

Outcome measurement bias varies by outcome type. Objective measures, such as implant stability assessed by ISQ or Periotest, were predominantly rated as low risk, whereas subjective outcomes, especially postoperative pain, more frequently presented some concerns due to limited assessor blinding. Selective reporting was judged as some concerns in several trials owing to unavailable protocols and inconsistencies in outcome reporting.

Publication bias was further explored using funnel plot inspection for all outcomes. Quantitative evaluation using Egger’s regression test was performed strictly for outcomes with at least ten included studies (such as implant stability assessed by ISQ), which did not indicate significant publication bias (*p* > 0.05). For outcomes with fewer than ten trials—specifically postoperative pain, marginal bone loss, and peri-implant bone density—quantitative regression tests were not conducted to avoid false-negative findings due to low statistical power. Instead, publication bias for these parameters was evaluated qualitatively via visual inspection of funnel plot symmetry, which revealed a generally symmetrical distribution of studies, indicating a low likelihood of serious publication bias.

In summary, while the overall methodological quality of the included trials was acceptable, recurring limitations related to allocation concealment, blinding, incomplete reporting, and limited power to detect publication bias were considered when interpreting pooled estimates and informing the GRADE certainty assessment.

### 3.6. Certainty of Evidence (GRADE) (Table 3 and Supplementary Table S7)

The certainty of evidence varied across outcomes according to the GRADE framework. For implant stability assessed by ISQ, the certainty was rated as low, reflecting imprecision (as several subgroup analyses were non-significant) and serious inconsistency. Although the statistical heterogeneity was moderate (*I*^2^ = 37%), the downgrade for inconsistency was driven by substantial clinical and methodological diversity across the included trials (specifically, variations in laser wavelengths, application protocols, and implant designs) rather than the statistical index alone. Similarly, Periotest outcomes were classified as low certainty, since although the overall estimate favored PBM, the effect was not consistently observed over time. For peri-implant bone density, due to the physical and mathematical incompatibility of pooling different measurement scales, the certainty of evidence was evaluated separately by imaging modality. Both the 3D CBCT (Hounsfield Units) and 2D conventional radiography (grayscale) subgroups were rated as having very low certainty. This classification was driven by serious risk of bias, serious imprecision due to small cumulative sample sizes and wide confidence intervals, and serious-to-very-serious inconsistency resulting from extensive clinical and protocol diversity. Notably, the time-stratified 2D conventional radiography subgroup captured a modest but statistically significant overall benefit favoring PBM (MD = 9.82; 95% CI: 1.08 to 18.56; *p* = 0.03), while the 3D CBCT subgroup overall estimate was non-significant due to extreme chronological heterogeneity across follow-up intervals (MD = 86.02 HU; 95% CI: −28.48 to 200.52; *p* = 0.14), despite demonstrating a robust, statistically significant bone density increase specifically at the baseline measurement (MD = 114.07 HU; *p* = 0.01). For marginal bone loss, the certainty was also low, as the observed reduction favoring PBM was small and unlikely to be clinically meaningful, in addition to moderate variability across studies. In contrast, postoperative pain demonstrated moderate certainty of evidence, with consistent findings and no observed heterogeneity; however, the magnitude of the effect was modest, which may limit its clinical relevance. Overall, while PBM offers notable benefits, particularly in pain reduction, early stability, and bone density, the evidence regarding some peri-implant tissue outcomes remains limited by methodological variability and should be interpreted with caution (Table 3).

**Table 3:** Summary of findings (GRADE) for primary outcomes.

Outcome	N° of RCTs	Effect (PBM vs. Control)	Risk of Bias	Inconsistency	Imprecision	Publication Bias	Certainty
Implant stability (ISQ)	13	MD = 1.26; 95% CI 0.61 to 1.91; favors PBM	Some concerns	Serious (Clinical diversity; *I*^2^ = 37%)	Serious (wide CIs in subgroups)	Possible	Low
Implant stability (Periotest)	5	MD = −0.55; 95% CI −0.84 to −0.27; favors PBM	Some concerns	Serious (*I*^2^ = 67%)	Not serious	Unlikely	Low
Bone density (3D CBCT/Hounsfield Units)	3	MD = 86.02 HU; 95% CI: −28.48 to 200.52; *p* = 0.14 (non-significant)	Some concerns ^1^	Very Serious ^2^ (*I*^2^ = 99%)	Serious ^3^ (wide CIs)	Possible ^4^	Very Low
Bone density (2D radiography/Grayscale)	2	MD = 9.82; 95% CI: 1.08 to 18.56; *p* = 0.03 (favors PBM)	Some concerns ^1^	Serious ^2^ (*I*^2^ = 67%)	Serious ^3^ (small sample)	Possible ^4^	Very Low
Marginal bone loss (MBL)	6	MD = −0.09 mm; 95% CI −0.16 to −0.01; favors PBM	Some concerns	Serious (*I*^2^ = 58%)	Serious (small effect size)	Possible	Low
Postoperative pain (VAS)	6	MD = −0.27; 95% CI −0.35 to −0.19; favors PBM	Some concerns	Not serious (*I*^2^ = 0%)	Serious (small effect size)	Unlikely	Moderate

Notes: ^1^ Risk of Bias: Downgraded by one level due to methodological limitations in some of the included trials, specifically regarding a lack of details on allocation concealment and the blinding of outcome assessors (RoB 2). ^2^ Inconsistency: Downgraded due to high statistical heterogeneity *I*^2^ = 99% for the 3D CBCT subgroup, evaluated as very serious; and *I*^2^ = 67% for the 2D conventional radiography subgroup, evaluated as serious) and substantial clinical variability in PBM parameters—including variations in wavelengths (635 to 940 nm), energy densities, and implant characteristics across the trials. ^3^ Imprecision: Downgraded by one level because of small cumulative sample sizes and wide confidence intervals. Specifically, the 3D CBCT subgroup overall confidence interval crosses the line of no effect (null value; *p* = 0.14), while the 2D conventional radiography subgroup is limited by a small sample size (only 2 included trials). ^4^ Publication Bias: Publication bias was evaluated qualitatively via visual inspection of funnel plots. Quantitative regression tests (such as Egger’s regression) were not performed because the number of trials in each subgroup was less than ten (N < 10), in strict accordance with the Cochrane Handbook guidelines. Background colors indicate the GRADE certainty of evidence categories: high, moderate, low, and very low certainty.

## 4. Discussion

This systematic review and meta-analysis synthesized evidence from 32 randomized controlled trials evaluating the effects of PBM on implant stability, peri-implant bone outcomes, and postoperative pain in adults undergoing dental implant surgery. Overall, the clinical evidence demonstrates that PBM provides valuable adjunctive benefits, assisting in the reduction of postoperative pain, enhancing both functional (Periotest) and structural (ISQ) early implant stability, and promoting accelerated peri-implant bone density development. These findings are highly consistent with the known biological effects of PBM on cellular metabolism [3], osteoblastic activity [12], angiogenesis [13], and inflammatory modulation [10], suggesting its potential role as a promising supportive intervention in implant dentistry.

Implant stability assessed via ISQ demonstrated a statistically significant overall improvement favoring the PBM group (MD = 1.26; 95% CI 0.61 to 1.91; *p* < 0.001), indicating that implant sites treated with PBM achieved an average increase of 1.26 ISQ points compared to control sites. This finding aligns with the early functional improvements detected by Periotest measurements (MD = −0.55; 95% CI −0.84 to −0.27; *p* = 0.0002), suggesting a synergistic effect of PBM in counteracting the physiological stability ‘dip’ typically observed during the early weeks of healing. However, the clinical relevance of this statistical significance warrants a cautious interpretation. While the overall pooled effect of 1.26 ISQ points favors PBM, the lack of independent statistical significance at most individual follow-up intervals indicates that PBM’s localized effect at specific weeks is subtle. The statistical significance observed in our meta-analysis is primarily enhanced by the cumulative statistical power of the pooled sample, rather than a massive clinical breakthrough at any single healing interval. Therefore, clinicians should view PBM as a supportive, adjunctive tool that promotes a subtle, cumulative acceleration of early stability, rather than a guarantee of significantly higher ISQ values at every specific postoperative week [27,34,35].

Complementing the ISQ findings, Periotest measurements also demonstrated a statistically significant overall effect favoring PBM, indicating a positive trend in early functional stability. This finding is supported by clinical studies reporting enhanced damping capacity and biomechanical behavior in the early healing phase, reflecting the sensitivity of Periotest to viscoelastic changes at the bone–implant interface. While resonance frequency analysis (ISQ) primarily reflects lateral structural stiffness and bone-to-implant contact rigidity, Periotest evaluates damping capacity and micromobility, which are highly sensitive to early cellular and biochemical changes in the peri-implant environment [32,33,37,47,49]. Rather than presenting a discrepancy, the joint analysis of these two methods suggests that PBM helps mitigate the temporary reduction in stability during early bone remodeling by accelerating biological attachment at the interface.

In contrast, peri-implant bone density outcomes demonstrated a clear divergence when analyzed strictly by imaging modality and stratified by follow-up intervals. In studies utilizing three-dimensional CBCT-derived Hounsfield Units (Figure 4a), the overall time-stratified pooled estimate showed a mean difference of 86.02 HU (95% CI: −28.48 to 200.52; *p* = 0.14; *I*^2^ = 99%); however, PBM promoted a highly robust, statistically significant bone density increase specifically at baseline (MD = 114.07 HU; 95% CI: 22.78 to 205.36; *p* = 0.01) [33,40,41]. Conversely, the two-dimensional (2D) conventional radiography subgroup (Figure 4b), which was corrected for baseline decimal reporting, demonstrated a modest but statistically significant overall benefit favoring PBM across the pooled time points (MD = 9.82; 95% CI: 1.08 to 18.56; *p* = 0.03; *I*^2^ = 67%), with a notable long-term mineralizing trend captured at the 6-month interval (MD = 24.19; 95% CI: 0.89 to 47.48; *p* = 0.04) [28,31]. This methodological distinction indicates that tomographic density assessments (HU) are highly sensitive for capturing immediate postoperative mineral changes, whereas 2D radiographic scales can successfully track cumulative bone density gains over longer healing periods.

Despite these biologically plausible effects, the findings regarding bone density should be interpreted with caution. Reflecting this clinical uncertainty, the certainty of evidence for both subgroups was graded as very low according to the GRADE framework. The extreme statistical heterogeneity observed in the 3D CBCT subgroup (*I*^2^ = 99%) underscores the significant chronological variability and protocol differences across the tomographic trials, particularly the temporal variations in bone remodeling. Even in the 2D conventional radiography subgroup, moderate-to-high heterogeneity remained (*I*^2^ = 67%), indicating that differences in laser parameters, patient-specific factors, and follow-up schedules limit direct comparability. Therefore, although PBM demonstrates positive trends—particularly at the 6-month interval—the substantial methodological variability across current studies warrants caution regarding the clinical generalizability of these bone density outcomes [28,31,33,40,41].

The clinical significance of PBM is hypothesized to be closely related to a physiological ‘catch-up’ effect. While PBM appears to provide notable biomechanical and mineralizing advantages during the critical early healing weeks—potentially mitigating the temporary stability ‘dip’ that typically occurs as primary mechanical stability transitions to secondary biological stability—these relative differences seem to narrow over longer follow-up periods [18]. Under this explanatory hypothesis, as bone remodeling progresses toward completion, the natural healing capacity of the control group may eventually catch up with the accelerated initial gains stimulated by PBM. Therefore, the primary clinical value of PBM is suggested to lie not in altering the final, long-term osseointegration endpoint, but rather in accelerating the early healing timeline, reducing patient discomfort, and minimizing early failure risks during the critical initial phases of implant placement. Future prospective longitudinal studies are warranted to directly test and map the long-term kinetics of this hypothesized convergence [59].

Postoperative pain reduction emerged as the most consistent finding of this study, with no observed heterogeneity and moderate certainty of evidence. PBM significantly reduced pain, particularly during the early postoperative period, which is consistent with its known anti-inflammatory and analgesic mechanisms [36,44,47,54,57,58]. Specifically, clinical evidence confirms that active laser biostimulation significantly minimizes immediate postoperative discomfort, edema, and rescue analgesic intake, thereby accelerating patient recovery in the critical first days following implant placement [55]. This clinical benefit is particularly valuable because poorly controlled acute postoperative pain can lead to increased stress and potentially transition into chronic symptoms. Although the absolute magnitude of this pain reduction was modest, PBM’s ability to consistently alleviate acute discomfort in the first 24 to 72 h represents a highly meaningful improvement in patient-centered outcomes and postoperative quality of life.

Similarly, MBL demonstrated a statistically significant reduction favoring PBM (MD = −0.09 mm; 95% CI −0.16 to −0.01; *p* = 0.02). While PBM may attenuate early crestal bone resorption by modulating local inflammatory cytokines and promoting microcirculation, the biological magnitude of this reduction—fractions of a millimeter—is relatively small. Indeed, according to the classic implant success criteria established by Albrektsson et al. (1986) [59], a marginal bone loss of up to 1.5 mm during the first year of clinical loading is considered acceptable, with subsequent bone loss restricted to less than 0.2 mm annually. Our observed pooled difference of 0.09 mm favoring the PBM group is substantially below these established clinical thresholds. Furthermore, from a diagnostic perspective, a difference of 0.09 mm falls within the standard measurement error and resolution limits of conventional intraoral radiographs (typically ranging between 0.1 mm and 0.2 mm). Consequently, while this small reduction in bone loss is statistically significant, it lacks clinical detectability or immediate clinical relevance on its own. However, when considered alongside the significant improvements in bone density and early stability, this small but consistent reduction in marginal bone loss supports the overall bone-preserving and regenerative influence of PBM at the peri-implant crestal interface during early healing [32,35,39,43,46,47,51,53].

The GRADE assessment further contextualizes these findings. The certainty of evidence ranged from moderate to very low, primarily due to risk of bias, inconsistency, and imprecision. For implant stability outcomes (ISQ and Periotest), certainty was low, reflecting clinical diversity, variability across time points, and wide confidence intervals. For peri-implant bone density, both the 3D CBCT and 2D radiographic subgroups were graded as very low certainty, reflecting serious risk of bias, protocol diversity, and wide confidence intervals across trials. MBL was also rated as low certainty, as the small magnitude of effect limits its clinical relevance on its own. In contrast, postoperative pain achieved moderate certainty due to highly consistent findings across studies, despite the modest effect size.

These results are consistent with previous systematic reviews, which have also reported heterogeneous and inconclusive evidence regarding the effectiveness of PBM in implant dentistry [19,20]. Although some studies suggest benefits in early healing and pain reduction, the lack of standardized protocols and consistent outcomes limits the strength of current evidence.

To address these limitations, future randomized controlled trials should adopt standardized and transparently reported PBM protocols to improve comparability and reproducibility. Based on current evidence, future studies should explore wavelengths within the red (630–699 nm) and near-infrared (780–980 nm) spectra, with defined ranges for power output, energy delivery, and fluence. In addition, secondary parameters—such as irradiance, spot size, exposure time, treatment geometry, and number of sessions—should be consistently reported. The use of combined intraoperative and early postoperative protocols, along with standardized follow-up intervals and validated outcome measures [48], may help reduce heterogeneity and strengthen evidence synthesis.

Furthermore, future research should prioritize well-designed and adequately powered randomized controlled trials, with rigorous methodology, including appropriate handling of clustered data and standardized outcome assessment. The incorporation of objective biomarkers and advanced imaging techniques—specifically, quantitative three-dimensional tomographic evaluations (Hounsfield Units) rather than conventional two-dimensional radiographic grayscale measurements—will substantially enhance the understanding of PBM effects on osseointegration. Multicenter collaborations [60] are also encouraged to improve external validity and support the development of consensus-based clinical guidelines.

## 5. Conclusions

Photobiomodulation serves as a promising supportive intervention in dental implant surgery. The synthesis of clinical evidence demonstrates that PBM consistently reduces early postoperative pain and significantly improves both early functional stability (Periotest MD = −0.55) and overall structural stability (ISQ MD = 1.26; *p* < 0.001). Furthermore, when evaluated using time-stratified models, PBM promotes a robust, statistically significant initial bone density increase in 3D CBCT-derived Hounsfield Units at baseline (MD = 114.07 HU; *p* = 0.01; overall MD = 86.02 HU; *p* = 0.14), while conventional 2D radiographic grayscale measurements capture a modest but statistically significant overall density benefit across the pooled time points (MD = 9.82; *p* = 0.03), showing a positive long-term mineralizing trend up to 6 months (MD = 24.19; *p* = 0.04). This is accompanied by a modest but significant reduction in marginal bone loss (MD = −0.09 mm; *p* = 0.02). Consequently, while the overall certainty of evidence according to the GRADE framework ranges from low to very low, PBM can be suggested as a helpful adjunctive option to improve patient comfort, accelerate stability recovery, and optimize early bone mineralization in implant dentistry, although clinicians should interpret these findings with caution due to the substantial chronological and methodological heterogeneity across protocols.

## Data Availability

The original contributions presented in this study are included in the article and supplementary materials. Further inquiries can be directed to the corresponding author.

## Supplementary Materials

The following supporting information can be downloaded at: https://www.mdpi.com/article/10.3390/dj14090599/s1, Table S1: PRISMA 2020 Checklist for systematic reviews and meta-analysis; Table S1A: PRISMA-P Checklist; Table S2: Complete Search Strategies; Table S3: Characteristics of Included Studies; Table S4: PBM Protocols; Table S5: Outcomes and Study-Level Findings; Table S6A–E: Extracted Data for Meta-analysis; Figure S1: Risk of Bias Assessment (RoB 2); Figure S2A–F: Forest Plots; Figure S3A–E: Funnel Plots for Publication Bias; Table S7A: GRADE Evidence Profiles; Table S7B: Justifications for Downgrading and Upgrading Decisions (GRADE); Table S8A–C: Standardized and Recommended PBM Parameters; Table S9: Recommendations For Future PBM Clinical Trials In Implant Dentistry.

## Abbreviations

The following abbreviations are used in this manuscript:

PBM	Photobiomodulation
LLLT	Low-Level Laser Therapy
LED	Light-Emitting Diode
ISQ	Implant Stability Quotient
MBL	Marginal Bone Loss
RCT	Randomized Controlled Trial
PRISMA	Preferred Reporting Items for Systematic Reviews and Meta-Analyses
GRADE	Grading of Recommendations Assessment, Development and Evaluation
RoB 2	Risk of Bias 2
HU	Hounsfield Units
MD	Mean Difference
CI	Confidence Interval
CBCT	Cone Beam Computed Tomography
VAS	Visual Analog Scale
NRS	Numeric Rating Scale
ATP	Adenosine Triphosphate
