# Supplementary material for: Photobiomodulation in Dental Implant Surgery: A Systematic Review and Meta-Analysis of Randomized Controlled Trials
Source: Dent J (Basel). 2026 Sep 16;14(9):599. doi: 10.3390/dj14090599 (PMC13604971; doi:10.3390/dj14090599)
Supplement: Supplementary file 1 [file dentistry-14-00599-s001.zip › dentistry-4437608-supplementary.pdf]

SUPPLEMENTARY MATERIAL
for
PBM in Dental Implants: A Meta-Analysis

Table of Contents

- **Supplementary Table S1.** PRISMA 2020 Checklist
- **Supplementary Table S1A.** PRISMA-P Checklist
- **Supplementary Table S2.** Complete Search Strategies
- **Supplementary Table S3.** Characteristics of Included Studies
- **Supplementary Table S4.** PBM Protocols
- **Supplementary Table S5.** Outcomes and Study-Level Findings
- **Supplementary Table S6A.**Extracted Data for Meta-analysis: ISQ
- **Supplementary Table S6B.**Extracted Data for Meta-analysis:Periotest
- **Supplementary Table S6C.**Extracted Data for Meta-analysis: Bone density
- **Supplementary Table S6D.**Extracted Data for Meta-analysis: Marginal Bone Loss
- **Supplementary Table S6E.**Extracted Data for Meta-analysis: Pain VAS/NRS
- **Supplementary Figure S1.** Risk of Bias Assessment (RoB 2)
- **Supplementary Figure S2A.** Forest Plot: ISQ
- **Supplementary Figure S2B.** Forest Plot: Periotest
- **Supplementary Figure S2C.** Forest Plot: Bone Density 3D CBCT-HU
- **Supplementary Figure S2D.** Forest Plot: Bone Density (2D) conventional radiographic
- **Supplementary Figure S2E.** Forest Plot: Marginal Bone Loss
- **Supplementary Figure S2F.** Forest Plot: Pain
- **Supplementary Figure S3A.** Funnel Plot for Publication Bias: ISQ
- **Supplementary Figure S3B.** Funnel Plot for Publication Bias: Periotest
- **Supplementary Figure S3C.** Funnel Plot for Publication Bias: Bone Density
- **Supplementary Figure S3D.** Funnel Plot for Publication Bias: Marginal Bone Loss
- **Supplementary Figure S3E.** Funnel Plot for Publication Bias: Pain
- **Supplementary Table S7A.** Summary of Findings Table (GRADE Framework) Photobiomodulation in Dental Implant Therapy
- **Supplementary Table S7B.** Justifications for Certainty Downgrading (GRADE)
- **Supplementary Table S8A.** Standardized Photobiomodulation Parameters Across Included RCTs
- **Supplementary Table S8B.** Recommended Parameters for Future RCTs
- **Supplementary Table S8C.** Comparison Between Observed PBM Parameters in Included RCTs (S11) and Recommended Parameters for Future RCTs (S11b)
- **Supplementary Table S9.** Recommendations For Future PBM Clinical Trials In Implant Dentistry

SUPPLEMENTARY TABLE S1. PRISMA 2020 CHECKLIST FOR SYSTEMATIC REVIEWS AND META-ANALYSIS

This table provides the completed PRISMA 2020 checklist for the present systematic review and meta-analysis, indicating where each reporting item is addressed within the manuscript.

Supplementary Table S1. PRISMA 2020 CHECKLIST

Section / Topic	PRISMA Item	Checklist Description	Location in Manuscript
**Title**	1	Identify the report as a systematic review	Title page
**Abstract**	2	Structured summary of background, methods, results, and conclusions	Abstract
**Introduction**	3	Rationale for the review	Introduction, paragraphs 1-6
	4	Objectives or questions addressed	End of Introduction
**Methods**	5	Eligibility criteria	Methods 2.3; Table 1
	6	Information sources	Methods 2.2
	7	Search strategy	Methods 2.2; Supplementary Material S2
	8	Selection process	Methods 2.1 and 2.5
	9	Data collection process	Methods 2.5
	10	Data items	Methods 2.3; 2.5; Table2
	11	Study risk of bias assessment	Methods 2.6
	12	Effect measures	Methods 2.7
	13a	Synthesis methods – meta-analysis models	Methods 2.3 and 2.8
	13b	Heterogeneity assessment	Methods 2.5 and 2.7
	13c	Data transformation or handling	Methods 2.7
	13d	Sensitivity/subgroup analyses	Methods 2.7
	13e	Heterogeneity	Methods 2.8
	13f	Sensitivity analyses	Methods 2.8
	14	Reporting bias assessment	Methods 2.6 and 2.7
	15	Certainty assessment (GRADE)	Methods 2.9
**Results**	16a	Study selection results	Results 3.1; Figure 1
	16b	Excluded studies with reasons	Results 3.1
	17	Study characteristics	Results 3.2; Supplementary Table S3-S5
	18	Risk of bias	Results 3.5; Supplementary Figure S1
	19	Results of individual studies	Results 3.4; Figure2-6; Supplementary Materials Figures S2 and S3
	20a	Results of syntheses	Results 3.4
	20b	Heterogeneity and subgroup exploration	Results 3.4; Figure 2-6
	20c	Causes of heterogeneity	Results 3.4.1; 3.4.3; 2.8
	20d	Sensitivity analyses	Results 3.4.3 and 3.5

Section / Topic	PRISMA Item	Checklist Description	Location in Manuscript
	21	Reporting biases	Results 3.5; Supplementary Materials Figures S2 and S3
	22	Certainty of evidence (GRADE)	Results 3.6; Table 3; Supplementary Table S7
**Discussion**	23a	General interpretation	Discussion, paragraphs 1-10
	23b	Limitations of evidence	Discussion, paragraphs 6-10
	23c	Limitations of review process	Discussion, paragraphs 9-10
	23d	Implications for practice and research	Discussion, final paragraphs
**Other**	24a	Registration and protocol	Methods 2.1
	24b	Registration and protocol	Methods 2.1
	24c	Registration and protocol	Not applicable
	25	Support/funding	Funding; Acknowledgements
	26	Competing interests	Conflict of Interest
	27	Availability of data/materials	Supplementary Materials Tables S2-S6, S7 and Figures S1-S3

SUPPLEMENTARY TABLE S1A. PRISMA-P CHECKLIST

Section / Topic	Item No.	Checklist Item	Reported (Yes/No)	Location in Manuscript / Supplementary Material
**Title**	1a	Identify the report as a systematic review and meta-analysis	Yes	Title
	1b	If an update, identify as such	Not applicable	—
**Registration**	2	Provide registration information	Yes	Methods – Study design; PROSPERO CRD420251041607
**Authors**	3a	Provide author contact details	Yes	Separate Title Page
	3b	Describe contributions of authors	Yes	Author Contributions section
**Amendments**	4	Describe protocol amendments	No amendments	Methods
**Support**	5a	Describe sources of financial support	Yes	Funding
	5b	Provide sponsor name	Yes	Funding (CAPES – Finance Code 001)
	5c	Describe role of funder	Yes	Funding
**Rationale**	6	Describe rationale for the review	Yes	Introduction
**Objectives**	7	Provide explicit statement of objectives	Yes	Introduction (PICO)
**Eligibility criteria**	8	Specify inclusion and exclusion criteria	Yes	Methods – Eligibility Criteria
**Information sources**	9	Describe information sources	Yes	Methods – Search Strategy
**Search strategy**	10	Present full search strategy	Yes	Supplementary Material S2
**Data management**	11a	Describe data management	Yes	Methods – Data Extraction
**Selection process**	11b	Describe study selection process	Yes	Methods – Study Selection
**Data collection process**	11c	Describe data extraction process	Yes	Methods – Data Extraction
**Data items**	12	List and define all data items	Yes	Methods; Supplementary Table S4
**Outcomes & prioritization**	13	List and define outcomes	Yes	Methods – Outcomes
**Risk of bias**	14	Describe risk of bias assessment	Yes	Methods; Supplementary Figure S1
**Data synthesis**	15a	Describe criteria for quantitative synthesis	Yes	Methods – Statistical Analysis
	15b	Describe summary measures and synthesis methods	Yes	Methods – Statistical Analysis
	15c	Describe additional analyses	Yes	Methods; Results
	15d	Describe narrative synthesis	Yes	Results
**Meta-bias(es)**	16	Describe assessment of publication bias	Yes	Supplementary Material Figure S3
**Confidence in evidence**	17	Describe assessment of certainty of evidence	Yes	Results – GRADE; Table 3; Supplementary Table S7

SUPPLEMENTARY TABLE S2. COMPLETE SEARCH STRATEGIES

Supplementary Table S2. Database search strategies used for the identification of randomized controlled trials.

This table presents the full electronic search strings applied across all scientific databases and grey literature sources, ensuring reproducibility and transparency of the systematic review process.

1. PubMed / MEDLINE Search Strategy
Database: PubMed (NCBI)
Date of last search: May 2025

("photobiomodulation" OR "low level laser therapy" OR "low-level laser therapy"
OR "LLLT" OR "laser therapy" OR "LED therapy" OR "light emitting diode"
OR "near infrared" OR "red light therapy")
AND
("dental implants" OR "implant dentistry" OR "osseointegration"
OR "oral implant" OR "implant stability")
AND
("randomized controlled trial" OR "randomised controlled trial"
OR "controlled clinical trial" OR "RCT" OR "split mouth")
NOT
(animal OR mice OR rat OR canine OR in vitro)

Filters applied:

- Humans
- Clinical Trial / Randomized Controlled Trial
- English

2. EMBASE (Elsevier) Search Strategy
Database: EMBASE (Ovid)
Date of last search: May 2025

('photobiomodulation'/exp OR 'photobiomodulation' OR 'low level laser therapy'/exp
OR 'low level laser therapy' OR 'lllt' OR 'laser therapy'/exp
OR 'light emitting diode' OR 'led therapy')
AND
('dental implant'/exp OR 'dental implant' OR 'osseointegration'/exp
OR 'osseointegration' OR 'implant stability')
AND
('randomized controlled trial'/exp OR 'clinical trial'/exp OR randomized OR randomised)
NOT
('animal'/exp OR 'in vitro study'/exp)

Filters:

- Humans
- Randomized Controlled Trial

3. Scopus (Elsevier) Search Strategy
Database: Scopus
Date of last search: May 2025

TITLE-ABS-KEY("photobiomodulation" OR "low-level laser therapy" OR "LLLT"
OR "laser phototherapy" OR "LED therapy")
AND
TITLE-ABS-KEY("dental implant" OR "implant stability" OR "osseointegration")
AND
TITLE-ABS-KEY("randomized controlled trial" OR "split-mouth" OR "parallel trial")
AND NOT TITLE-ABS-KEY("in vitro" OR "animal study" OR "rat" OR "mouse")

Filters:

- Article
- English
- Clinical Trials

4. Cochrane Library / CENTRAL Search Strategy
Database: Cochrane Central Register of Controlled Trials (CENTRAL)
Date of last search: May 2025

(photobiomodulation OR "low level laser" OR lllt OR "laser therapy" OR "led therapy")
AND
("dental implant" OR osseointegration OR "implant stability")
IN Trials

No filters beyond CENTRAL's automated RCT indexing.

5. ScienceDirect Search Strategy
Database: ScienceDirect (Elsevier)
Date of last search: May 2025

("photobiomodulation" OR "low level laser therapy" OR "LLLT" OR "laser therapy")
AND
("dental implant" OR "implant stability" OR "osseointegration")
AND
("randomized" OR "controlled trial" OR "clinical trial")

Filters:

- Research articles
- Medicine & Dentistry
- 2000–2025 (automatic default range)

6. Google Scholar (Grey Literature)
Approach: Google Scholar does not allow Boolean strings as precise as PubMed.
Search string used:

photobiomodulation dental implant randomized controlled trial

Procedure:

- First 200 results screened (sorted by relevance, as recommended in PRISMA).
- Titles and abstracts screened for eligibility.
- Duplicates removed.

7. Manual Search Strategy
Journals manually screened:
- Lasers in Medical Science
- Photodiagnosis and Photodynamic Therapy
- Photomedicine and Laser Surgery
- Journal of Oral Implantology
- Clinical Oral Implants Research
- International Journal of Implant Dentistry
- Journal of Periodontology

Procedure:
- Screening of *last 5 years* issues (2020–2025).
- Screening of reference lists of all included studies and previous systematic reviews.

8. ResearchGate & Grey Sources
Used for:
- Conference papers
- Preprints
- Unpublished PBM datasets
- Dissertation-like materials on PBM and implants

Search terms: “photobiomodulation implant stability”, “LLLT osseointegration RCT”.
Only studies that met full RCT criteria and provided extractable data were considered.

SUPPLEMENTARY TABLE S3 – CHARACTERISTICS OF INCLUDED STUDIES

Supplementary Table S3. Study and participant characteristics of the included randomized controlled trials.

This table summarizes the country, design, sample size, demographic profile, implant sites, implant systems and insertion torque reported in each randomized controlled trial included in the review.

Study Ref	Study (year)	Country	Study design	Patients (n)	Implants (n)	Sex (M:F)	Age (years)	Implant site(s)	Implant system / brand	Insertion torque	Notes
R23	García-Morales et al., 2012	Brazil	Split-mouth RCT	8	30	2:6	20–55	Bilateral posterior mandible	XiVE-S (Dentsply)	Not reported	Healed sites
R24	Hamed et al., 2013	Egypt	Parallel RCT	11	11	9:2	22–40	Anterior maxilla	Not reported (Spectra System)	Manual insertion	Immediate implants
R41	Mandic et al., 2015	Serbia	Split-mouth RCT	12	40	6:6	≥18	Bilateral posterior maxilla	BlueSky (Bredent)	15 rpm / 35 Ncm	Self-drilling implants
R46	Memarian et al., 2018	Iran	Split-mouth RCT	12	36	Not reported	Not reported	Anterior mandible	DIO (DIO Corp.)	Not reported	Same diameter and length in both groups
R25	Torkzaban et al., 2018	Iran	Parallel RCT	19	80	9:10	mean 40.8 / 43.0	Maxilla (mainly posterior, low-density bone)	DIO	25–35 Ncm	4.0 or 4.5 × 10–11.5 mm
R26	Karaca et al., 2017	Turkey	Split-mouth RCT	25	100	11:14	36–64	Posterior mandible	DTI implants	35–50 Ncm	Immediate loading
R27	Mikhail et al., 2018	Egypt	Parallel RCT	20	20	Not reported	30–40	Not specified (maxilla/mandible)	Dentium	35 Ncm	4.0–4.5 × 10–12 mm
R28	Elsyad et al., 2019	Egypt	Split-mouth RCT	20	40	Not reported	55–65	Mandibular canine region (overdenture mini-implants)	Mini Plus implants (Cowellmedi)	40 Ncm	Type 2 diabetics with immediate loading
R29	Matys et al., 2019	Poland	Parallel RCT	24	40	16:8	46.7 ± 8.7	Posterior mandible	Superline (Daegu)	Not reported	Healed sites
R42	Lobato et al., 2019	Brazil	Parallel RCT	44	50	Not reported	20–79	Anterior and posterior (maxilla and mandible)	Neodent (Straumann group)	15–60 Ncm	Fresh extraction sockets
R43	El Makaky et al., 2020	Egypt	Split-mouth RCT	40	40	9:11	≥18	Bilateral posterior maxilla	IMPLA System (Schutz Dental)	30–35 Ncm	13–14.5 mm, 4.5–5.3 mm diameter

R30	Bozkaya et al., 2021	Turkey	Split-mouth RCT	20	93	5:15	55.6 ± 12.0	Maxilla (15, 16, 25, 26)	Straumann Standard	30–50 Ncm	Healed sites
R31	Kinalski et al., 2021	Brazil	Parallel RCT	32	63	Not reported	49.9 ± 11.3	Not specified (mostly mandible)	Straumann	10–70 Ncm	Healed sites
R32	Reza et al., 2021	Iran	Split-mouth RCT	23	63	12:9	46.8	Posterior mandible	SNUCONE (SNUCONE Co.)	Not reported	Healed sites
R33	Bilge et al., 2022	Turkey	Split-mouth RCT	22	66	17:16	51.5 ± 6.9	Maxilla and mandible	Not specified	35 Ncm	Partially edentulous patients
R45	Alashhab et al., 2021	Syria	Split-mouth RCT	12	24	Not reported	Not reported	Bilateral posterior maxilla	Megapen Implants	35 Ncm	Healed sites
R47	Camolesi et al., 2023	Spain	Parallel RCT	13	40	5:8	Not reported	Maxilla and mandible	Ti IV – Model IPX (Nueva Galimplant)	Not reported	Single-tooth and short-span restorations
R34	Fahim et al., 2022	Egypt	Parallel RCT	12	20	Not reported	22–45	Posterior mandible	Not reported	35 Ncm	Delayed placement
R48	Gokmenoglu et al., 2014	Turkey	Parallel RCT	15	22	9:6	Not reported	Partially edentulous jaws	XiVE-S (Dentsply)	Not reported	Healed sites
R44	Palled et al., 2021	India	Parallel RCT	30	30	13:17	>18	Fully edentulous mandible (overdentures)	Not reported	Not reported	Mandibular overdenture implants
R35	Bakry et al., 2020	Egypt	Parallel RCT	12	12	Not reported	30–50	Maxillary premolar region	SwissPlus (Zimmer)	Not reported	Healed sites
R36	Arakeeb et al., 2019	Egypt	Parallel RCT	40	40	40:0	30–40	Posterior mandible	TiXos	Not reported	Male patients only
R49	Gulati et al., 2020	India	Parallel RCT	20	20	Not reported	Not reported	Mandible	Touareg-S (Adin)	Not reported	Healed sites
R37	Attia et al., 2023	Egypt	Parallel RCT	40	40	21:18	40–48	Posterior jaws	Multysystem (Lissome)	Not reported	Type 2 diabetics
R38	Momeni et al., 2024	Iran	Parallel RCT	10	10	3:7	41.3	Maxilla and/or mandible	AnyRidge (MegaGen)	Not reported	Healed sites
R50	Basualdo Allende et al., 2024	Chile	Split-mouth RCT	20	20	10:10	Not reported	Edentulous areas in maxilla and mandible	Not reported	Not reported	PBM vs. Sham placebo, (0 J)
R51	Caccianiga et al., 2020	Italy	Parallel RCT	60	60	27:33	47.13 ± 8.05	Edentulous regions in maxilla and mandible	Not reported	Not reported	Three-arm study (laser vs placebo vs control)

Study ID	Study (year)	Country	Study design	Patients (n)	Implants (n)	Sex (M:F)	Age (years)	Implant site(s)	Implant system / brand	Insertion torque	Notes
R39	Deljavan et al., 2024	Iran	Parallel RCT	30	30	20:10	46.7	Maxilla and mandible	Not specified	Not reported	Healed sites
R52	Mohajerani et al., 2020	India	Parallel RCT	58	58	32:26	Not reported	Mandibular first molar region	Zimmer	Not reported	4.8 × 10 mm implants
R53	Zou et al., 2024	China	Parallel RCT	58	70	28:30	Not reported	Not reported	Not reported	Not reported	Data partially missing
R40	Collado Murcia et al., 2024	Spain	Parallel RCT	62	Not reported*	36:26	53.5	Maxilla and mandible	Straumann	Not reported	Healed sites
R54	Makker et al., 2024	India	Split-mouth RCT	5	10	Not reported	Not reported	Mandible	Not reported	Not reported	Small pilot trial

Note:
* Collado Murcia et al. (2024) did not report the exact total number of implants, only patient distribution (18 patients with 1 implant and 13 patients with >1 implant in the PBM group; 21 patients with 1 implant and 10 patients with >1 implant in the Sham group).

SUPPLEMENTARY MATERIAL S4 – PBM PROTOCOLS

Supplementary Table S4. PBM parameters and treatment protocols in the included randomized controlled trials.

This table summarizes the type of device, wavelength, emission mode, dose, application protocol, number of sessions, treatment schedule and comparator used in each trial.

Study ID	Study (year)	PBM device / type	Wavelength(s) (nm)	Emission mode	Dose / fluence (approx.)	Application protocol (time, points, energy)	No. of sessions	Treatment schedule	Comparator
R23	García-Morales et al., 2012	GaAlAs diode laser	830	Continuous	≈92.1 J/cm²	3 s per point, 20 points, 5 J per session	7	Every 48 h for 14 days (post-operative)	Placebo
R24	Hamed et al., 2013	Diode laser	980	Continuous	Not reported (500 mW output)	5 min per session	8	Twice per week for 1 month	Control (no PBM)
R41	Mandic et al., 2015	GaAlAs diode laser	637	Continuous	6.26 J/cm², 40 mW	3 irradiation points per session	7	Once daily for 7 consecutive days	Control
R46	Memarian et al., 2018	Diode + LED	810 (laser) + 626 (LED)	Not stated / continuous	50 mW, 20 J/cm² (laser); 20 mW (LED)	400 s (laser) and 20 min (LED) per session	6	Days 0, 3, 7, 10 and 14 (combined PBM)	Control
R25	Torkzaban et al., 2018	Diode laser	940	Continuous	354.6 mW/cm²; 8 J per point	40 s per point; 2 points; 100 mW	7	Every 2 days until day 12 post-surgery	Control
R26	Karaca et al., 2017	GaAlAs diode laser	830	Continuous	≈92.1 J/cm²	20 points, 5 J per session	5	Days 0, 1, 3, 5 and 7	Ozone therapy and non-irradiated control
R27	Mikhail et al., 2018	GaAlAs diode laser	904	Continuous	4.7 J/cm²	30 s, 20 mW per application	9	Three applications per day (with 1 h interval) on postoperative days 2, 4 and 6	Control
R28	Elsyad et al., 2019	GaAlAs diode laser	940	Continuous	9 J/cm²	60 s per application, 6 irradiation points	3	Immediately after surgery, day 3 and 1 week	Control
R29	Matys et al., 2019	Red diode laser	635	Continuous	8 J/cm²	100 mW; 4 J/cm² per point; 2 points; 40 s	6	1 day before surgery, immediately after, and on days 2, 4, 7 and 14	Control
R42	Lobato et al., 2019	GaAlAs diode laser	808	Continuous	66 J/cm² (≈11 J/cm² per point)	6 points, total time ≈1.23 min	2	In the socket after extraction and again after suturing	Control
R43	El Makaky et al., 2020	AlGaInP diode laser	660	Continuous	40.96 J/cm²	40 s per point, 2 points, 75 mW	8	Immediately post-op and on days 2, 4, 6, 8, 10, 12 and 14	Control
R30	Bozkaya et al., 2021	GaAlAs diode laser	830	Continuous	42 J/cm²	3 s per point; 20 points; 0.3 J per point	7	Immediately after surgery, then 3×/week for 2 weeks	Control
R31	Kinalski et al., 2021	GaAlAs diode laser	808	Continuous	66 J/cm² (11 J/cm² per point)	6 points; 80 s; 50 mW	2	In the socket before implant placement and immediately after	Control

Study ID	Study (year)	PBM device / type	Wavelength(s) (nm)	Emission mode	Dose / fluence (approx.)	Application protocol (time, points, energy)	No. of sessions	Treatment schedule	Comparator
R32	Reza et al., 2021	GaAlAs + InGaAlP lasers	808 + 660	Pulsed	12 J/cm² total	6 J over 3 points, 20 s at 400 mW (808 nm) and 40 mW for 200 s (660 nm)	2	Immediately after surgery and 48 h later	Placebo
R33	Bilge et al., 2022	InGaAsP diode laser	940	Continuous	≈50 J/cm²	4 points; 200 s; ≈40 J per session	6	Immediately post-op; days 3, 7, 14; 1 and 2 months	Placebo
R45	Alashhab et al., 2021	GaAlAs diode laser	808	Continuous	8 J/cm²	4 J/cm² per point; 2 points; 16 s	7	Immediately post-op and on days 2, 4, 6, 8, 10 and 12	Control
R47	Camolesi et al., 2023	Diode laser	808 + 630	Continuous	66 J/cm²	10 J per session; 100 s; 3 points	2	Immediately after surgery and at 7 days	Placebo
R34	Fahim et al., 2022	Diode laser	980	Continuous	60 J/cm²	30 J over 2 points	6	3 days pre-surgery; after extraction; after implant placement; additional post-operative sessions over 1 week	Control
R48	Gokmenoglu et al., 2014	LED array	626	Continuous	222 J/cm² (≈46.2 J/cm² per emitter)	20 min per session; 8 light emitters	10	Immediately post-op and on days 2, 4, 6, 8, 10, 12, 14, 16 and 18	Control
R44	Palled et al., 2021	Nd:YAG laser	1064	Pulsed	28 J/cm² (≈7.07 J/cm² per application)	2 J; 30 Hz; 60 s along mesio-distal aspect	4	Immediately post-op, at weeks 2 and 6, and at 3 months	Control
R35	Bakry et al., 2020	Diode laser	980	Continuous	≈321.4 J/cm² (≈21.4 J/cm² per session)	2 W; 300 Hz; 5 min per application	15	1 session 2 weeks before surgery, then 2 sessions every 2 weeks up to 6 months	Control
R36	Arakeeb et al., 2019	GaAlAs diode laser	808	Continuous	120 J/cm² (20 J/cm² per point)	1 min per application; 2 points	6	Immediately after surgery and on days 2, 4, 6, 8, 10 and 12	Control
R49	Gulati et al., 2020	GaAlAs diode laser	980	Continuous	24 J/cm²	6 points; 6 J per session	4	Immediately post-op and on days 3, 7 and 14	Control
R37	Attia et al., 2023	GaAlAs diode laser	808	Continuous	66 J/cm² (11 J/cm² per point)	6 points; ≈1.23 min	2	After implant placement and after suturing	Control
R38	Momeni et al., 2024	GaAlAs diode laser	940	Continuous	90 J/cm² (5 J/cm² per point)	18 points; 10 s per point	1	Single session immediately after surgery	Control
R50	Basualdo Allende et al., 2024	InGaAlP + AlGaAs lasers	660 + 808	Continuous	22.5 J/cm² (4.5 J/cm² per point)	5 points per session	1	Single post-operative session	Placebo (0J)
R51	Caccianiga et al., 2020	LED device	450–835	Continuous	45.1 J/cm²	730 s per session	1	Single post-operative session	PBM vs placebo vs control
R39	Deljavan et al., 2024	LED device	660	Continuous	6.26 J/cm²	1 point; 400 mW per session	7	Immediately post-op and on days 2, 4, 6, 8, 10 and 12	Control

Study ID	Study (year)	PBM device / type	Wavelength(s) (nm)	Emission mode	Dose / fluence (approx.)	Application protocol (time, points, energy)	No. of sessions	Treatment schedule	Comparator
R52	Mohajerani et al., 2020	LED + GaAlAs laser	632 + 830	Continuous	≈480.7 J/cm²	4 points; 20 min per session	10	Immediately after surgery and for 9 consecutive days	Placebo
R53	Zou et al., 2024	Nd:YAG laser	1064	Continuous	≈1080 J/cm² (180 J/cm² per point)	2 points per session	3	Immediately post-op and on days 2 and 3	Control
R40	Collado Murcia et al., 2024	GaAlAs diode laser	940	Continuous	45 J/cm² (15 J/cm² per point)	30 s per point; 3 points	1	Single post-operative session	Placebo
R54	Makker et al., 2024	GaAlAs diode laser	810	Continuous	20 J/cm² (5 J/cm² per point)	2 points; 10 s per point	2	Immediately after surgery and on day 3	Placebo

Abbreviations: *PBM, photobiomodulation; LLLT, low-level laser therapy; LED, light-emitting diode; Nd:YAG, neodymium-doped yttrium aluminum garnet; J, joule; J/cm², joules per square centimetre; mW, milliwatt; W, watt; Hz, hertz.*

SUPPLEMENTARY MATERIAL S5 – OUTCOMES AND STUDY-LEVEL FINDINGS

Supplementary Table S5. Summary of outcome measures, follow-up, main findings and study limitations of the included randomized controlled trials.

This table provides an overview of the primary outcomes assessed in each study, the duration of follow-up, authors' main conclusions, and reported methodological limitations.

Study Ref	Study (year)	Outcome(s) measured	Time points	Follow-up	Main findings	Study limitations
R23	García-Morales et al., 2012	ISQ (RFA)	10 days; 3, 6, 9, 12 weeks	6 months	No effect of LLLT on implant stability measured by RFA.	Small sample size.
R24	Hamed et al., 2013	Bone density (Digora)	Immediate; 2 weeks; 1, 3, 6 months	6 months	Significant positive effect on bone healing and density.	Small sample; only immediate implants; needs evaluation in different bone types.
R41	Mandic et al., 2015	ISQ & ALP activity	Weekly measures	6 weeks	Self-drilling implants showed high early success, regardless of LLLT.	Small sample; short follow-up; implant design may have masked PBM effects.
R46	Memarian et al., 2018	Periotest	Immediate; days 3, 7, 10, 14	8 weeks	PBM and LED groups showed higher stability at week 3.	Small sample; short follow-up.
R25	Torkzaban et al., 2018	ISQ	Immediate; day 10; weeks 3, 6, 12	12 weeks	No significant effect of 940 nm PBM in low-density maxillary bone.	Small sample; high primary stability may obscure PBM effects.
R26	Karaca et al., 2017	ISQ	Immediate; 6 months	6 months	PBM and ozone increased stability in immediate mandibular implants.	Specific implant type and anatomical area limit generalization.
R27	Mikhail et al., 2018	Bone density (Digora)	Immediate; 1.5 months; 6 months	6 months	PBM enhanced osseointegration; combination with Omega-3, Vitamin C, and Calcium improved outcomes.	No explicit limitations stated by authors.
R28	Elsyad et al., 2019	Periotest, CBCT	Immediate; 6 and 12 months	12 months	PBM improved implant stability at 6 months, but not marginal bone loss.	Small sample; only diabetic patients; immediate loading.
R29	Matys et al., 2019	Periotest, CBCT	Immediate; 2, 4 weeks; 2, 3 months	3 months	Improved secondary stability and bone density at 12 weeks.	Small sample; short follow-up.
R42	Lobato et al., 2019	ISQ	Immediate; 4–6 months	4–6 months	No effect of PBM on implant stability during healing.	Single application; implant heterogeneity.
R43	El Makaky et al., 2020	Periotest	Immediate; 6 and 12 months	12 months	No significant difference in stability or marginal bone loss.	High baseline stability.
R30	Bozkaya et al., 2021	ISQ	Immediate; 30, 60, 90 days	3 months	No significant effect on stabilization.	Small sample; limited follow-up duration.
R31	Kinalski et al., 2021	ISQ & radiographic bone	Immediate; 4 and 6 months	6 months	PBM did not improve stability in healed sites.	Lack of standardization in bone quality.
R32	Reza et al., 2021	ISQ, pain, wound healing	14 days	2 weeks	PBM (dual wavelength) improved healing and reduced postoperative pain.	Unable to isolate effects of each wavelength.
R33	Bilge et al., 2022	Periotest	Immediate; 14 days; 1 and 2 months	12 months	PBM enhanced osseointegration and secondary stability.	Small sample; lack of standardized parameters.

Study Ref	Study (year)	Outcome(s) measured	Time points	Follow-up	Main findings	Study limitations
R45	Alashhab et al., 2021	ISQ	Immediate; 7 days; 3 months	3 months	PBM increased early stability, but effect not sustained at 3 months.	Small sample; high primary stability.
R47	Camolesi et al., 2023	ISQ, soft-tissue healing	Immediate; day 7; weeks 4 and 8	8 weeks	No long-term stability effect; improved soft-tissue healing.	No ISQ data at weeks 2 and 8; implant diameter variation.
R34	Fahim et al., 2022	ISQ	6-week secondary stability endpoint	6 months	PBM significantly improved secondary stability and soft-tissue healing.	Small sample.
R48	Gokmenoglu et al., 2014	ISQ	Immediate; 2, 4, 6, 12 weeks	3 months	PBM group maintained stability; control group showed decline.	Small sample; smokers not excluded.
R44	Palled et al., 2021	ISQ	2 and 6 weeks; 3 months	3 months	PBM improved peri-implant healing and osseointegration.	Small sample; short follow-up.
R35	Bakry et al., 2020	Bone density & MBL	Immediate; 2, 3, 6 months	6 months	No significant effect on hard or soft tissue around implants.	Small sample; protocol may not be optimal.
R36	Arakeeb et al., 2019	CBCT density	Immediate; day 7; weeks 6 and 12	3 months	PBM + L-PRF synergistically improved osseointegration.	Only male participants; need wider parameter testing.
R49	Gulati et al., 2020	MBL, soft-tissue healing	Immediate; days 3, 7, 14	2 weeks	PBM reduced marginal bone resorption.	Small sample.
R37	Attia et al., 2023	Bone density, biomarkers (OPG)	Immediate; 6 months	6 months	PBM increased bone density in Type II diabetic patients.	Only single intraoperative session; authors suggest extended protocols may enhance results.
R38	Momeni et al., 2024	ISQ	Immediate; 12 weeks	3 months	PBM significantly improved vestibulo-lingual secondary stability.	Small sample; single PBM session.
R50	Basualdo Allende et al., 2024	ISQ	Immediate; follow-up not detailed	Not reported	Laser vs LED PBM showed differing responses; early improvements noted.	Limited reporting; small sample.
R51	Caccianiga et al., 2020	MBL	Immediate; 6 months	6 months	PBM groups showed reduced bone loss compared to placebo/control.	Single session may limit interpretation.
R39	Deljavan et al., 2024	MBL	Immediate; 6 months	6 months	PBM reduced bone resorption compared to control.	Small sample.
R52	Mohajerani et al., 2020	ISQ	Immediate; 10 days; 3, 6, 9 weeks	9 weeks	Combined LED + laser improved stability at 9 weeks.	Short follow-up duration.
R53	Zou et al., 2024	Pain (VAS), soft-tissue healing	Days 1–3	2 weeks	PBM reduced pain and enhanced early soft-tissue healing.	Small sample.
R40	Collado Murcia et al., 2024	Pain (VAS), inflammation	24 h; 48 h; day 7	1 week	Pain and inflammation decreased similarly with or without PBM.	Subjective pain reporting; lack of stability measurements.
R54	Makker et al., 2024	Pain (VAS)	12 h; 24 h; 48 h; 72 h	3 days	PBM reduced intensity and duration of postoperative pain.	Very small sample.

SUPPLEMENTARY TABLE S6A — EXTRACTED DATA FOR META-ANALYSIS: ISQ

Supplementary Table S6A. Quantitative ISQ data extracted from randomized controlled trials, grouped by time point.

PBM = photobiomodulation group; CTRL = control group; SD = standard deviation.

Time point	Study Ref	Study (year)	PBM Mean	PBM SD	PBM n	CTRL Mean	CTRL SD	CTRL n
**Immediate**	R45	Alashhab et al., 2021	75.00	4.156	12	75.25	3.388	12
	R47	Camolesi et al., 2023	71.50	8.8	20	71.90	5.9	20
	R23	García-Morales et al., 2012	77.40	3.40	15	75.70	5.60	14
	R48	Gokmenoglu et al., 2014	73.20	7.90	10	77.90	4.80	12
	R26	Karaca et al., 2017	73.00	3.25	25	73.00	3.13	25
	R31	Kinalski et al., 2021	62.02	16.41	22	61.36	20.02	30
	R42	Lobato et al., 2019	59.84	12.90	25	59.29	13.41	25
	R41	Mandic et al., 2015	76.00	3.52	22	72.89	7.15	22
	R52	Mohajerani et al., 2020	67.26	6.80	28	68.00	6.42	28
	R38	Momeni et al., 2024	69.20	3.46	5	72.30	3.86	5
	R25	Torkzaban et al., 2018	58.50	6.19	40	58.62	6.30	40
	R30	Bozkaya et al., 2021	74.00	10.00	47	71.50	11.20	46
**7 days**	R41	Mandic et al., 2015	74.88	3.40	22	74.69	4.80	22
	R45	Alashhab et al., 2021	76.50	3.580	12	73.83	2.443	12
**10 days**	R23	García-Morales et al., 2012	78.90	3.70	15	76.20	4.60	14
	R52	Mohajerani et al., 2020	68.24	6.51	28	64.71	6.91	28
	R25	Torkzaban et al., 2018	54.87	6.92	40	54.12	7.63	40
**2 weeks**	R41	Mandic et al., 2015	74.22	3.93	22	72.56	5.67	22
	R48	Gokmenoglu et al., 2014	71.80	5.20	10	69.30	7.40	12
	R44	Palled et al., 2021	57.93	3.95	15	35.67	3.08	15
**3 weeks**	R23	García-Morales et al., 2012	76.8	4.6	15	76.9	3.5	14
	R41	Mandic et al., 2015	72.67	3.65	22	70.44	6.16	22
	R25	Torkzaban et al., 2018	54.55	6.75	40	53.87	7.59	40
	R52	Mohajerani et al., 2020	68.56	6.55	28	63.26	6.21	28
**4 weeks**	R41	Mandic et al., 2015	72.50	4.18	22	69.22	9.09	22
	R48	Gokmenoglu et al., 2014	69.30	5.50	10	68.70	8.20	12
	R30	Bozkaya et al., 2021	74.00	11.50	47	72.50	12.00	46
**6 weeks**	R23	García-Morales et al., 2012	75.5	4.0	15	76.3	2.3	14

Time point	Study Ref	Study (year)	PBM Mean	PBM SD	PBM n	CTRL Mean	CTRL SD	CTRL n
	R41	Mandic et al., 2015	72.67	3.69	22	70.61	7.20	22
	R25	Torkzaban et al., 2018	55.35	6.88	40	54.42	7.44	40
	R34	Fahim et al., 2022	71.89	2.67	10	62.43	8.62	10
	R44	Palled et al., 2021	67.80	2.48	15	52.40	3.15	15
	R52	Mohajerani et al., 2020	70.52	6.14	28	64.92	6.34	28
**9 weeks**	R23	García-Morales et al., 2012	76.2	4.9	15	77.7	3.5	14
	R52	Mohajerani et al., 2020	71.18	5.75	28	66.50	6.73	28
**12 weeks**	R23	García-Morales et al., 2012	76.3	4.1	15	78.4	3.0	14
	R25	Torkzaban et al., 2018	57.9	6.34	40	55.85	6.82	40
	R45	Alashhab et al., 2021	70.83	4.609	12	68.16	4.877	12
	R48	Gokmenoglu et al., 2014	71.4	4.2	10	71.7	6.0	12
	R44	Palled et al., 2021	67.06	3.78	15	58.87	3.75	15
	R38	Momeni et al., 2024	73.80	6.34	5	74.10	5.60	5
	R30	Bozkaya et al., 2021	80.00	9.00	47	76.00	12.50	46
**6 months**	R26	Karaca et al., 2017	79.0	2.51	25	77.0	2.14	25
	R31	Kinalski et al., 2021	62.90	11.19	22	65.12	8.67	30
	R42	Lobato et al., 2019	68.11	12.06	25	64.19	10.60	25

SUPPLEMENTARY TABLE S6B — EXTRACTED DATA FOR META-ANALYSIS: PERIOTEST

Supplementary Table S6B. Quantitative Periotest values extracted from randomized controlled trials, grouped by time point (lower values indicate greater stability).
PBM = photobiomodulation group; CTRL = control group; SD = standard deviation.

Time point	Study Ref	Study (year)	PBM Mean	PBM SD	PBM n	CTRL Mean	CTRL SD	CTRL n
**Immediate**	R46	Memarian et al., 2018	-5.52	1.380	12	-6.12	1.019	12
	R28	Elsyad et al., 2019	-2.00	0.86	20	-1.12	0.49	20
	R29	Matys et al., 2019	-5.37	0.52	18	-5.35	0.68	22
	R43	El Makaky et al., 2020	-2.05	0.759	20	-1.95	0.826	20
	R33	Bilge et al., 2022	-3.9182	2.938	33	-4.0242	2.638	33
**2 weeks**	R29	Matys et al., 2019	-5.30	0.46	18	-4.48	0.53	22
	R33	Bilge et al., 2022	-4.2091	2.776	33	-3.3212	2.843	33
**4 weeks**	R46	Memarian et al., 2018	-5.98	1.052	12	-4.58	1.294	12
	R29	Matys et al., 2019	-4.64	0.51	18	-4.04	0.59	22
	R33	Bilge et al., 2022	-4.5864	2.540	33	-3.2636	2.664	33
**8 weeks**	R46	Memarian et al., 2018	-6.68	1.159	12	-5.46	1.264	12
	R29	Matys et al., 2019	-5.01	0.41	18	-4.63	0.59	22
**12 weeks**	R29	Matys et al., 2019	-5.53	0.55	18	-4.35	2.89	22
	R33	Bilge et al., 2022	-6.0909	2.268	33	-3.848	2.817	33

SUPPLEMENTARY TABLE S6C — EXTRACTED DATA FOR META-ANALYSIS: PAIN VAS/NRS

Supplementary Table S6C. Patient-reported postoperative pain measured by VAS or NRS scales. Lower scores indicate less pain. Data grouped by time point.
PBM = photobiomodulation group; CTRL = control group; SD = standard deviation.

Time point	Study Ref	Study (year)	PBM Mean	PBM SD	PBM n	CTRL Mean	CTRL SD	CTRL n
**Immediate**	R26	Karaca et al., 2017	3.0	1.43	25	3.0	1.43	25
	R43	Makaky et al., 2020	3.45	1.099	20	3.50	0.946	20
	R32	Reza et al., 2021	3.14	1.10	21	3.48	1.12	21
**Day 1**	R26	Karaca et al., 2017	4.9	2.03	25	4.9	2.03	25
	R43	Makaky et al., 2020	5.00	1.124	20	5.20	0.834	20
	R32	Reza et al., 2021	1.9	1.0	21	2.2	1.0	21
	R53	Zou et al., 2024	4.0	3.0	35	4.0	3.0	35
	R40	Collado Murcia et al., 2024	2.07	2.72	31	2.10	2.31	31
	R54	Makker et al., 2024	3.8	0.45	5	4.6	0.55	5
**Day 2**	R53	Zou et al., 2024	3.0	2.0	35	3.0	3.0	35
	R40	Collado Murcia et al., 2024	1.97	2.75	31	1.65	1.02	31
	R54	Makker et al., 2024	2.40	0.55	5	3.40	0.55	5
**Day 3**	R26	Karaca et al., 2017	4.0	1.72	25	4.0	1.70	25
	R43	Makaky et al., 2020	4.70	1.081	20	4.80	0.894	20
	R32	Reza et al., 2021	0.9	0.8	21	1.2	0.9	21
	R53	Zou et al., 2024	2.0	1.0	35	2.0	2.0	35
	R54	Makker et al., 2024	1.00	0.71	5	2.0	0.71	5
**Day 5**	R26	Karaca et al., 2017	2.6	1.16	25	2.6	1.16	25
	R43	Makaky et al., 2020	2.85	0.875	20	2.95	0.999	20
	R32	Reza et al., 2021	0.3	0.1	21	0.6	0.2	21
**Day 7**	R26	Karaca et al., 2017	1.8	0.85	25	1.8	0.85	25
	R43	Makaky et al., 2020	1.45	0.605	20	1.55	0.759	20
	R40	Collado Murcia et al., 2024	0.40	0.86	31	0.61	1.02	31

SUPPLEMENTARY TABLE S6D — EXTRACTED DATA FOR META-ANALYSIS: BONE DENSITY

Supplementary Table S6D. Radiographic bone density outcomes extracted for quantitative synthesis. Higher values indicate greater bone density. Data grouped by time point.
PBM = photobiomodulation group; CTRL = control group; SD = standard deviation.

Time point	Study Ref	Study (year)	PBM Mean	PBM SD	PBM n	CTRL Mean	CTRL SD	CTRL n
**Immediate**	R24	Hamed et al., 2013	96.7	7.10	6	97.7	4.70	5
	R29	Matys et al., 2019	1177.44	96.19	18	1014.14	46.30	20
	R27	Mikhail et al., 2018	129.02	9.29	9	127.08	18.60	9
	R37	Attia et al., 2023	896.00	28.68	20	826.00	44.47	20
**4 weeks**	R24	Hamed et al., 2013	130.3	12.7	6	125.0	10.0	5
	R29	Matys et al., 2019	598.39	30.78	18	659.78	36.54	20
**6 weeks**	R27	Mikhail et al., 2018	139.53	11.43	9	132.06	19.57	9
	R36	Arakeeb et al., 2019	597.987	23.9	10	618.16	24.7	10
**12 weeks**	R24	Hamed et al., 2013	144.7	18.2	6	129.0	14.1	5
	R29	Matys et al., 2019	811.69	75.48	18	391.17	30.70	20
	R36	Arakeeb et al., 2019	640.379	21.8	10	690.632	25.5	10
**6 months**	R24	Hamed et al., 2013	173.0	14.1	6	136.3	15.2	5
	R37	Attia et al., 2023	960.00	22.71	20	888.25	92.64	20
	R27	Mikhail et al., 2018	150.76	9.86	9	137.86	18.81	9

SUPPLEMENTARY TABLE S6E — EXTRACTED DATA FOR META-ANALYSIS: MARGINAL BONE LOSS (MBL)

Supplementary Table S6E. Marginal bone loss (mm) at multiple follow-up periods. Positive values indicate greater bone loss. *PBM = photobiomodulation group; CTRL = control group; SD = standard deviation.*

Time point	Study Ref	Study (year)	PBM Mean	PBM SD	PBM n	CTRL Mean	CTRL SD	CTRL n
**Immediate**	R42	Lobato et al., 2019	3.86	1.54	25	4.07	1.96	25
	R43	Makaky et al., 2020	0.250	0.0827	20	0.255	0.0887	20
	R31	Kinalski et al., 2021	2.05	1.32	23	1.64	1.28	27
	R35	Bakry et al., 2020	2.40	0.60	6	2.40	1.30	6
	R49	Gulati et al., 2020	0.00	0.00	10	0.00	0.00	10
**12 weeks**	R35	Bakry et al., 2020	3.00	1.10	6	3.10	1.70	6
	R49	Gulati et al., 2020	0.81	0.13	10	0.97	0.09	10
**6 months**	R28	Elsyad et al., 2019	0.31	0.1443	20	0.4925	0.3172	20
	R42	Lobato et al., 2019	2.59	1.56	25	3.21	1.74	25
	R31	Kinalski et al., 2021	1.95	1.16	23	1.28	0.95	27
	R35	Bakry et al., 2020	2.80	1.00	6	3.10	1.30	6
**12 months**	R28	Elsyad et al., 2019	0.49	0.1818	20	0.715	0.3884	20
	R43	Makaky et al., 2020	0.265	0.0745	20	0.275	0.0716	20
	R49	Gulati et al., 2020	0.88	0.16	10	1.04	0.13	10

SUPPLEMENTARY FIGURE S1 — RISK OF BIAS ASSESSMENT (RoB 2)

Judgments per domain for all included randomized controlled trials.

Study ID	Experimental	Comparator	Outcome	Weight	D1	D2	D3	D4	D5	Overall
Garcia-Morales, 2012	Laser	Placebo	ISQ	1	+	+	+	+	+	+
Hamed et al., 2013	Laser	Control	Bone Density	1	-	+	+	+	+	!
Mandic et al., 2015	Laser	Control	ISQ and PICF	1	+	+	+	+	+	+
Memarian et al., 2018	Laser	Control	Periotest	1	+	+	+	+	!	!
Torkzaban et al., 2018	Laser	Placebo	ISQ	1	+	+	+	+	+	+
Karaca et al.,2017	Laser	Control	ISQ and VAS	1	!	!	+	+	!	!
Mikhail et al., 2018	Laser	Control	Bone Density	1	+	+	+	+	!	!
Elsyad et al., 2019	Laser	Control	Periotest and MBL	1	!	+	+	+	!	!
Matys et al., 2019	Laser	Control	Periotest and Bone Density	1	+	+	+	+	!	!
Lobato et al., 2019	Laser	Control	ISQ	1	+	+	+	+	!	!
Makaky et al., 2020	Laser	Control	Periotest and MBL	1	+	+	+	+	!	!
Kinalski et al., 2021	Laser	Control	ISQ and MBL	1	+	+	+	+	+	+
Reza et al., 2021	Laser	Placebo	VAS	1	+	+	+	+	!	!
Bilge et al., 2022	Laser	Placebo	Periotest and Bone Density	1	!	!	+	-	!	-
Alashhab, et al.,2021	Laser	Control	ISQ	1	!	!	+	-	!	-
Camolesi et al., 2023	Laser	Placebo	ISQ	1	+	+	+	+	+	+
Fahim, et al.,2022	Laser	Control	ISQ	1	!	!	-	-	!	-
Gokmenogly et al., 2014	LED	Control	ISQ and PICF	1	!	!	+	-	!	-
Palled et al., 2021	Laser	Control	ISQ and GCF	1	!	!	+	-	!	-
Bakry et al., 2020	Laser	Control	MBL, PD and DIM	1	+	!	+	+	!	!
Arakeeb et al., 2019	Laser	Control	Bone Density	1	+	!	+	+	!	!
Attia et al., 2023	Laser	Control	Bone Density and PICF	1	+	!	+	+	!	!
Momeni et al., 2024	Laser	Control	ISQ	1	!	!	+	-	!	-
Caccianiga et al., 2020	Laser	Control	NRS	1	+	+	+	+	+	+
Dejavan et al., 2024	LED	Control	MBL	1	+	+	+	+	+	+
Mohajerani, et al., 2020	Laser and LED	Placebo	ISQ	1	!	!	+	+	+	!
Zou, et al., 2024	Laser	Control	VAS, mSBI e GI	1	!	!	+	+	+	!
Collado Murcia, et al., 2024	Laser	Placebo	VAS	1	!	!	+	+	+	!
Makker, et al., 2024	Laser	Control	VAS	1	!	!	+	-	!	-
Gulati et al., 2020	Laser	Control	MBL	1	!	!	-	+	+	-
Allende et al., 2024	Laser	Placebo	VAS	1	+	+	+	+	!	!
Bozkaya et al., 2021	Laser	Control	ISQ and Microbiota	1	+	+	+	+	!	!

+ Low risk
! Some concerns
- High risk

D1 Randomisation process
D2 Deviations from the intended interventions
D3 Missing outcome data
D4 Measurement of the outcome
D5 Selection of the reported result

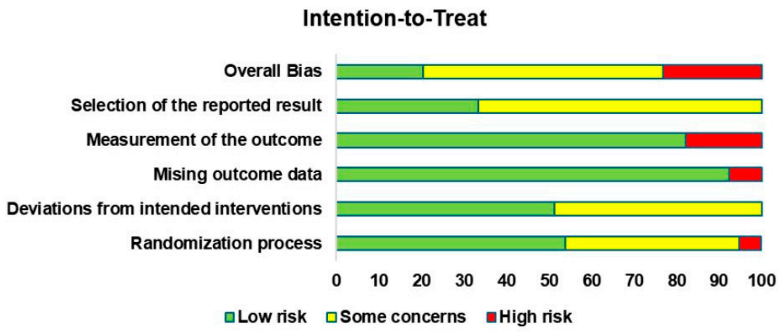

In RoB 2, the domain “bias due to deviations from intended interventions” incorporates considerations related to adherence, co-interventions, and intention-to-treat (ITT) principles. Studies that did not apply a true ITT approach or failed to account for protocol deviations were judged as “some concerns” in this domain.

Intention-to-treat (ITT): Analyses were performed according to the ITT principle, meaning that all randomized participants were included in the outcome assessment as originally allocated, irrespective of whether they completed the intervention, deviated from the protocol, or had missing follow-up data. This ensures preservation of the prognostic balance achieved by randomization.

SUPPLEMENTARY S2 — FORES PLOTS

Supplementary Figure S2. Forest plots summarizing the pooled effect sizes for each outcome included in the meta-analysis. Individual study estimates are shown with their corresponding 95% confidence intervals, and pooled effects were calculated using random-effects models. Heterogeneity was assessed using the I² statistic. Negative values indicate effects favoring photobiomodulation unless otherwise specified for each outcome.

S2A — Implant Stability: ISQ

Supplementary Figure S8A. Forest plot for implant stability measured by ISQ across all time points. The pooled estimate reflects early postoperative improvement associated with PBM, with diminishing differences at later stages.

	PBM			Control				Mean Difference
Study or Subgroup	Mean	SD	Total	Mean	SD	Total	Weight	IV, Random, 95% CI
1.1.1 *Baseline Implant Stability*								
Alashhab et al, 2021	75	4.156	12	75.25	3.388	12	2.8%	-0.25 [-3.28, 2.78]
Bozkaya et al, 2021	75	10	47	75	11	46	1.7%	0.00 [-4.28, 4.28]
Camolesi et al, 2023	71.5	8.8	20	71.9	5.9	20	1.5%	-0.40 [-5.04, 4.24]
Garcia-Morales, 2012	77.4	3.4	15	75.7	5.6	14	2.4%	1.70 [-1.70, 5.10]
Gokmenogly et al, 2014	73.2	7.9	10	77.9	4.8	12	1.1%	-4.70 [-10.30, 0.90]
Karaca et al, 2017	73	3.25	25	73	3.13	25	4.7%	0.00 [-1.77, 1.77]
Kinalski et al, 2021	62.02	16.41	22	61.36	20.02	30	0.4%	0.66 [-9.26, 10.58]
Lobato et al, 2019	59.84	12.9	25	59.29	13.41	25	0.7%	0.55 [-6.74, 7.84]
Mandic et al, 2015	76	3.52	22	72.89	7.15	22	2.5%	3.11 [-0.22, 6.44]
Mohajerani et al, 2020	67.26	6.8	28	68	6.42	28	2.4%	-0.74 [-4.20, 2.72]
Momeni et al, 2023	69.2	3.46	5	72.3	3.86	5	1.6%	-3.10 [-7.64, 1.44]
Torkzaban et al, 2018	58.5	6.19	40	58.62	6.3	40	3.2%	-0.12 [-2.86, 2.62]
**Subtotal (95% CI)**			**271**			**279**	**25.1%**	**0.02 [-0.96, 1.00]**
Heterogeneity: Tau² = 0.00; Chi² = 9.08, df = 11 (P = 0.61); I² = 0%								
Test for overall effect: Z = 0.04 (P = 0.97)								
1.1.2 *7 days*								
Alashhab et al, 2021	76.5	3.58	12	73.83	2.443	12	3.6%	2.67 [0.22, 5.12]
Mandic et al, 2015	74.88	3.4	22	74.69	4.8	22	3.6%	0.19 [-2.27, 2.65]
**Subtotal (95% CI)**			**34**			**34**	**7.1%**	**1.43 [-1.00, 3.86]**
Heterogeneity: Tau² = 1.51; Chi² = 1.96, df = 1 (P = 0.16); I² = 49%								
Test for overall effect: Z = 1.15 (P = 0.25)								
1.1.3 *10 days*								
Garcia-Morales, 2012	78.9	3.7	15	76.2	4.6	14	2.8%	2.70 [-0.35, 5.75]
Mohajerani et al, 2020	68.24	6.51	28	64.71	6.91	28	2.3%	3.53 [0.01, 7.05]
Torkzaban et al, 2018	54.87	6.92	40	54.12	7.63	40	2.6%	0.75 [-2.44, 3.94]
**Subtotal (95% CI)**			**83**			**82**	**7.7%**	**2.27 [0.40, 4.13]**
Heterogeneity: Tau² = 0.00; Chi² = 1.44, df = 2 (P = 0.49); I² = 0%								
Test for overall effect: Z = 2.38 (P = 0.02)								
1.1.4 *2 weeks*								
Gokmenogly et al, 2014	71.8	5.2	10	69.3	7.4	12	1.2%	2.50 [-2.78, 7.78]
Mandic et al, 2015	74.22	3.93	22	72.56	5.67	22	3.0%	1.66 [-1.22, 4.54]
**Subtotal (95% CI)**			**32**			**34**	**4.2%**	**1.85 [-0.68, 4.38]**
Heterogeneity: Tau² = 0.00; Chi² = 0.07, df = 1 (P = 0.78); I² = 0%								
Test for overall effect: Z = 1.43 (P = 0.15)								
1.1.5 *3 weeks*								
Garcia-Morales, 2012	76.8	4.6	15	76.9	3.5	14	2.9%	-0.10 [-3.06, 2.86]
Mandic et al, 2015	72.67	3.65	22	70.44	6.16	22	2.9%	2.23 [-0.76, 5.22]
Mohajerani et al, 2020	68.56	6.55	28	63.26	6.21	28	2.5%	5.30 [1.96, 8.64]
Torkzaban et al, 2018	54.55	6.75	40	53.87	7.59	40	2.7%	0.68 [-2.47, 3.83]
**Subtotal (95% CI)**			**105**			**104**	**10.9%**	**1.95 [-0.31, 4.21]**
Heterogeneity: Tau² = 2.80; Chi² = 6.35, df = 3 (P = 0.10); I² = 53%								
Test for overall effect: Z = 1.69 (P = 0.09)								
1.1.6 *4 weeks*								
Bozkaya et al, 2021	77	7.5	47	76.5	10.5	46	2.1%	0.50 [-3.22, 4.22]
Gokmenogly et al, 2014	69.3	5.5	10	68.7	8.2	12	1.1%	0.60 [-5.16, 6.36]
Mandic et al, 2015	72.5	4.18	22	69.22	9.09	22	1.8%	3.28 [-0.90, 7.46]
**Subtotal (95% CI)**			**79**			**80**	**5.0%**	**1.51 [-0.99, 4.02]**
Heterogeneity: Tau² = 0.00; Chi² = 1.07, df = 2 (P = 0.59); I² = 0%								
Test for overall effect: Z = 1.19 (P = 0.24)								
1.1.7 *6 weeks*								
Fahim et al, 2022	71.89	2.67	10	62.43	8.62	10	1.1%	9.46 [3.87, 15.05]
Garcia-Morales, 2012	75.5	4	15	76.3	2.3	14	3.7%	-0.80 [-3.16, 1.56]
Mandic et al, 2015	72.67	3.69	22	70.61	7.2	22	2.4%	2.06 [-1.32, 5.44]
Mohajerani et al, 2020	70.52	6.14	28	64.92	6.34	28	2.6%	5.60 [2.33, 8.87]
Torkzaban et al, 2018	55.35	6.88	40	54.42	7.44	40	2.7%	0.93 [-2.21, 4.07]
**Subtotal (95% CI)**			**115**			**114**	**12.5%**	**2.95 [-0.15, 6.06]**
Heterogeneity: Tau² = 9.32; Chi² = 17.36, df = 4 (P = 0.002); I² = 77%								
Test for overall effect: Z = 1.86 (P = 0.06)								
1.1.8 *9 weeks*								
Garcia-Morales, 2012	76.2	4.9	15	77.7	3.5	14	2.7%	-1.50 [-4.58, 1.58]
Mohajerani et al, 2020	71.18	5.75	28	66.5	6.73	28	2.5%	4.68 [1.40, 7.96]
**Subtotal (95% CI)**			**43**			**42**	**5.3%**	**1.56 [-4.49, 7.62]**
Heterogeneity: Tau² = 16.46; Chi² = 7.24, df = 1 (P = 0.007); I² = 86%								
Test for overall effect: Z = 0.51 (P = 0.61)								
1.1.9 *12 weeks*								
Alashhab et al, 2021	70.83	4.609	12	68.16	4.877	12	2.1%	2.67 [-1.13, 6.47]
Bozkaya et al, 2021	81	9	47	80	8	46	2.4%	1.00 [-2.46, 4.46]
Garcia-Morales, 2012	76.3	4.1	15	78.4	3	14	3.3%	-2.10 [-4.70, 0.50]
Gokmenogly et al, 2014	57.9	6.34	40	55.85	6.82	40	3.0%	2.05 [-0.84, 4.94]
Momeni et al, 2023	73.8	6.34	5	74.1	5.6	5	0.7%	-0.30 [-7.71, 7.11]
Torkzaban et al, 2018	57.9	6.34	40	55.85	6.82	40	3.0%	2.05 [-0.84, 4.94]
**Subtotal (95% CI)**			**159**			**157**	**14.4%**	**0.89 [-0.78, 2.55]**
Heterogeneity: Tau² = 1.31; Chi² = 7.24, df = 5 (P = 0.20); I² = 31%								
Test for overall effect: Z = 1.04 (P = 0.30)								
1.1.10 *6 months*								
Karaca et al, 2017	79	2.51	25	77	2.14	25	5.6%	2.00 [0.71, 3.29]
Kinalski et al, 2021	62.9	11.19	22	65.12	8.67	30	1.1%	-2.22 [-7.83, 3.39]
Lobato et al, 2019	68.11	12.06	25	64.19	10.6	25	0.9%	3.92 [-2.37, 10.21]
**Subtotal (95% CI)**			**72**			**80**	**7.7%**	**1.65 [-0.53, 3.83]**
Heterogeneity: Tau² = 1.19; Chi² = 2.49, df = 2 (P = 0.29); I² = 20%								
Test for overall effect: Z = 1.48 (P = 0.14)								
**Total (95% CI)**			**993**			**1006**	**100.0%**	**1.26 [0.61, 1.91]**
Heterogeneity: Tau² = 1.50; Chi² = 64.67, df = 41 (P = 0.01); I² = 37%								
Test for overall effect: Z = 3.81 (P = 0.0001)								
Test for subgroup differences: Chi² = 8.86, df = 9 (P = 0.45), I² = 0%								

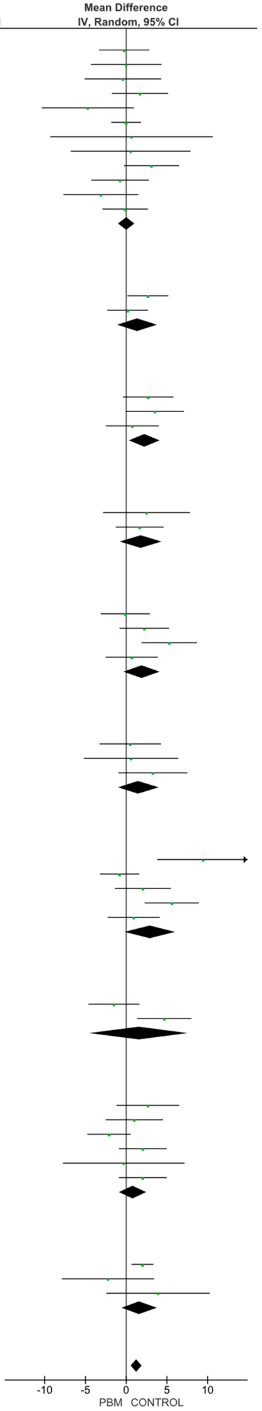

S2B — Implant Stability: Periotest

Supplementary Figure S2B. Forest plot for implant stability assessed by Periotest. Lower values indicate better stability. The pooled effect favors PBM in short-term assessments.

Study or Subgroup	Experimental Mean	Experimental SD	Experimental Total	Control Mean	Control SD	Control Total	Weight	Mean Difference IV, Random, 95% CI
**1.3.1 Baseline Implant Stability**								
Bilge et al, 2022	-3.9182	2.938	33	-4.0242	2.638	33	3.3%	0.11 [-1.24, 1.45]
Elsyad et al, 2019	-2	0.86	20	-1.12	0.49	20	9.2%	-0.88 [-1.31, -0.45]
Makaky et al, 2020	-2.05	0.759	20	-1.95	0.826	20	8.7%	-0.10 [-0.59, 0.39]
Matys et al, 2019	-5.37	0.52	18	-5.35	0.68	22	9.8%	-0.02 [-0.39, 0.35]
Memarian et al, 2018	-5.52	1.38	12	-6.12	1.019	12	5.0%	0.60 [-0.37, 1.57]
**Subtotal (95% CI)**			**103**			**107**	**35.9%**	**-0.16 [-0.64, 0.32]**
Heterogeneity: Tau² = 0.18; Chi² = 13.14, df = 4 (P = 0.01); I² = 70%								
Test for overall effect: Z = 0.66 (P = 0.51)								
**1.3.2 2 weeks**								
Bilge et al, 2022	-4.2091	2.776	33	-3.3212	2.843	33	3.2%	-0.89 [-2.24, 0.47]
Matys et al, 2019	1.81	3.2	22	1	0.66	21	3.2%	0.81 [-0.56, 2.18]
**Subtotal (95% CI)**			**55**			**54**	**6.4%**	**-0.04 [-1.71, 1.62]**
Heterogeneity: Tau² = 0.96; Chi² = 2.99, df = 1 (P = 0.08); I² = 67%								
Test for overall effect: Z = 0.05 (P = 0.96)								
**1.3.3 4 weeks**								
Bilge et al, 2022	-4.5864	2.54	33	-3.2636	2.664	33	3.6%	-1.32 [-2.58, -0.07]
Matys et al, 2019	-4.64	0.51	18	-4.04	0.59	22	10.0%	-0.60 [-0.94, -0.26]
Memarian et al, 2018	-5.98	1.052	12	-4.58	1.294	12	5.2%	-1.40 [-2.34, -0.46]
**Subtotal (95% CI)**			**63**			**67**	**18.8%**	**-0.92 [-1.49, -0.34]**
Heterogeneity: Tau² = 0.12; Chi² = 3.35, df = 2 (P = 0.19); I² = 40%								
Test for overall effect: Z = 3.13 (P = 0.002)								
**1.3.4 8 weeks**								
Matys et al, 2019	-5.01	0.41	18	-4.63	0.59	22	10.3%	-0.38 [-0.69, -0.07]
Memarian et al, 2018	-6.68	1.159	12	-5.46	1.264	12	5.0%	-1.22 [-2.19, -0.25]
**Subtotal (95% CI)**			**30**			**34**	**15.3%**	**-0.67 [-1.45, 0.11]**
Heterogeneity: Tau² = 0.22; Chi² = 2.61, df = 1 (P = 0.11); I² = 62%								
Test for overall effect: Z = 1.68 (P = 0.09)								
**1.3.5 12 weeks**								
Bilge et al, 2022	-6.0909	2.268	33	-3.848	2.817	33	3.7%	-2.24 [-3.48, -1.01]
Matys et al, 2019	-5.53	0.55	18	-4.35	2.89	22	3.7%	-1.18 [-2.41, 0.05]
**Subtotal (95% CI)**			**51**			**55**	**7.4%**	**-1.71 [-2.75, -0.67]**
Heterogeneity: Tau² = 0.17; Chi² = 1.43, df = 1 (P = 0.23); I² = 30%								
Test for overall effect: Z = 3.22 (P = 0.001)								
**1.3.7 6 months**								
Elsyad et al, 2019	-2.54	1.58	20	-1.34	0.42	20	6.8%	-1.20 [-1.92, -0.48]
Makaky et al, 2020	-3.25	0.639	20	-3.2	0.696	20	9.4%	-0.05 [-0.46, 0.36]
**Subtotal (95% CI)**			**40**			**40**	**16.1%**	**-0.59 [-1.71, 0.54]**
Heterogeneity: Tau² = 0.57; Chi² = 7.42, df = 1 (P = 0.006); I² = 87%								
Test for overall effect: Z = 1.02 (P = 0.31)								
**Total (95% CI)**			**342**			**357**	**100.0%**	**-0.55 [-0.84, -0.27]**
Heterogeneity: Tau² = 0.18; Chi² = 46.01, df = 15 (P < 0.0001); I² = 67%								
Test for overall effect: Z = 3.78 (P = 0.0002)								
Test for subgroup differences: Chi² = 9.20, df = 5 (P = 0.10), I² = 45.7%								

Mean Difference
IV, Random, 95% CI

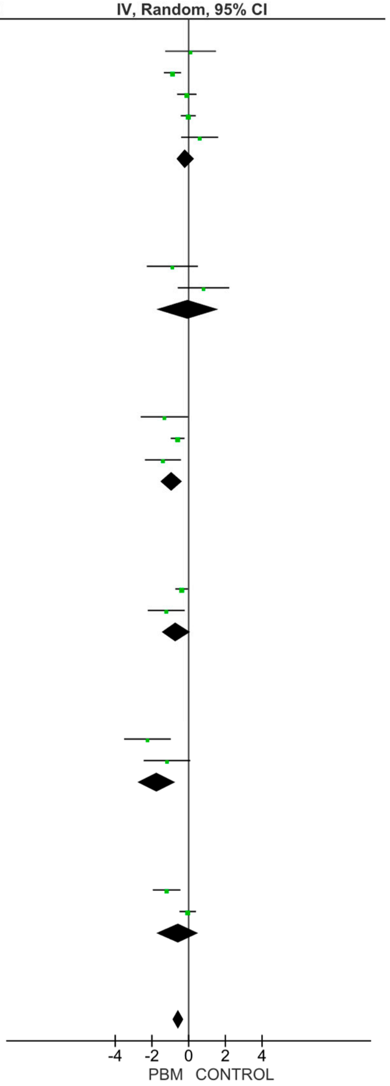

S2C — Bone Density 3D CBCT-HU

Supplementary Figure S2C. Forest plot for peri-implant bone density - 3D CBCT-derived Hounsfield Units (HU). Positive mean differences indicate greater bone density in the PBM group.

	PBM			Control				Mean Difference
Study or Subgroup	Mean	SD	Total	Mean	SD	Total	Weight	IV, Random, 95% CI
1.7.1 *Baseline Bone Density*								
Attia et al, 2023	896	28.68	20	826	44.47	20	16.8%	70.00 [46.81, 93.19]
Matys et al, 2019	1,177.44	96.19	18	1,014.14	46.3	20	16.4%	163.30 [114.45, 212.15]
**Subtotal (95% CI)**			**38**			**40**	**33.1%**	**114.07 [22.78, 205.36]**
Heterogeneity: Tau² = 3971.84; Chi² = 11.44, df = 1 (P = 0.0007); I² = 91%								
Test for overall effect: Z = 2.45 (P = 0.01)								
1.7.2 *4 weeks*								
Matys et al, 2019	598.39	30.78	18	659.78	36.54	20	16.8%	-61.39 [-82.81, -39.97]
**Subtotal (95% CI)**			**18**			**20**	**16.8%**	**-61.39 [-82.81, -39.97]**
Heterogeneity: Not applicable								
Test for overall effect: Z = 5.62 (P < 0.00001)								
1.7.3 *6 weeks*								
Arakeeb et al, 2019	597.987	23.9	10	618.16	24.7	10	16.8%	-20.17 [-41.48, 1.13]
**Subtotal (95% CI)**			**10**			**10**	**16.8%**	**-20.17 [-41.48, 1.13]**
Heterogeneity: Not applicable								
Test for overall effect: Z = 1.86 (P = 0.06)								
1.7.4 *12 weeks*								
Arakeeb et al, 2019	640.379	21.8	10	690.632	25.5	10	16.8%	-50.25 [-71.05, -29.46]
Matys et al, 2019	811.69	75.48	18	391.17	30.7	20	16.6%	420.52 [383.14, 457.90]
**Subtotal (95% CI)**			**28**			**30**	**33.3%**	**184.87 [-276.48, 646.22]**
Heterogeneity: Tau² = 110575.52; Chi² = 465.42, df = 1 (P < 0.00001); I² = 100%								
Test for overall effect: Z = 0.79 (P = 0.43)								
**Total (95% CI)**			**94**			**100**	**100.0%**	**86.02 [-28.48, 200.52]**
Heterogeneity: Tau² = 20232.52; Chi² = 605.00, df = 5 (P < 0.00001); I² = 99%								
Test for overall effect: Z = 1.47 (P = 0.14)								
Test for subgroup differences: Chi² = 18.78, df = 3 (P = 0.0003), I² = 84.0%								

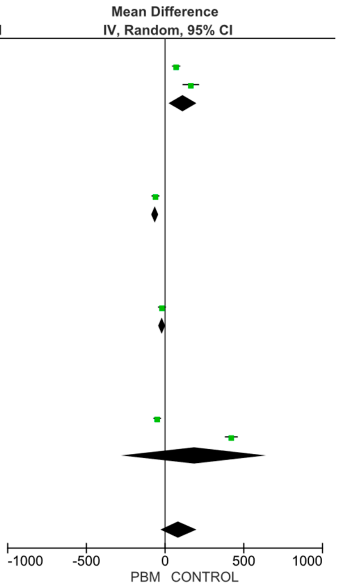

	PBM			Control				Mean Difference
Study or Subgroup	Mean	SD	Total	Mean	SD	Total	Weight	IV, Random, 95% CI
1.8.1 *Baseline bone density*								
Hamed et al, 2013	96.7	7.1	6	97.7	4.7	5	19.7%	-1.00 [-8.02, 6.02]
Mikhail et al, 2018	129.02	9.29	9	127.08	18.6	9	14.6%	1.94 [-11.64, 15.52]
**Subtotal (95% CI)**			**15**			**14**	**34.4%**	**-0.38 [-6.62, 5.85]**
Heterogeneity: Tau² = 0.00; Chi² = 0.14, df = 1 (P = 0.71); I² = 0%								
Test for overall effect: Z = 0.12 (P = 0.90)								
1.8.2 *4 weeks*								
Hamed et al, 2013	130.3	12.7	6	125	10	5	14.8%	5.30 [-8.12, 18.72]
**Subtotal (95% CI)**			**6**			**5**	**14.8%**	**5.30 [-8.12, 18.72]**
Heterogeneity: Not applicable								
Test for overall effect: Z = 0.77 (P = 0.44)								
1.8.3 *6 weeks*								
Mikhail et al, 2018	139.53	11.43	9	132.06	19.57	9	13.7%	7.47 [-7.34, 22.28]
**Subtotal (95% CI)**			**9**			**9**	**13.7%**	**7.47 [-7.34, 22.28]**
Heterogeneity: Not applicable								
Test for overall effect: Z = 0.99 (P = 0.32)								
1.8.4 *12 weeks*								
Hamed et al, 2013	144.7	18.2	6	129	14.1	5	10.9%	15.70 [-3.40, 34.80]
**Subtotal (95% CI)**			**6**			**5**	**10.9%**	**15.70 [-3.40, 34.80]**
Heterogeneity: Not applicable								
Test for overall effect: Z = 1.61 (P = 0.11)								
1.8.5 *6 months*								
Hamed et al, 2013	173	14.1	6	136.3	15.2	5	11.9%	36.70 [19.24, 54.16]
Mikhail et al, 2018	150.76	9.86	9	137.86	18.81	9	14.4%	12.90 [-0.97, 26.77]
**Subtotal (95% CI)**			**15**			**14**	**26.3%**	**24.19 [0.89, 47.48]**
Heterogeneity: Tau² = 218.49; Chi² = 4.38, df = 1 (P = 0.04); I² = 77%								
Test for overall effect: Z = 2.04 (P = 0.04)								
**Total (95% CI)**			**51**			**47**	**100.0%**	**9.82 [1.08, 18.56]**
Heterogeneity: Tau² = 87.96; Chi² = 18.03, df = 6 (P = 0.006); I² = 67%								
Test for overall effect: Z = 2.20 (P = 0.03)								
Test for subgroup differences: Chi² = 6.43, df = 4 (P = 0.17), I² = 37.8%								

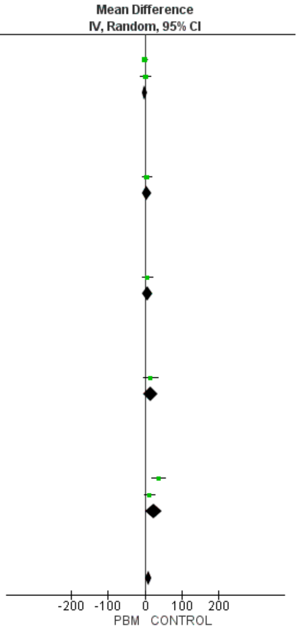

S8D — Bone Density (2D) conventional radiographic

Supplementary Figure S8D. Forest plot for peri-implant bone density - (2D) conventional radiographic. Positive mean differences indicate greater bone density in the PBM group.

S2E — Marginal Bone Loss (MBL)

Supplementary Figure S2E. Forest plot for marginal bone loss at follow-up. Negative values indicate reduced bone loss.

Study or Subgroup	PBM Mean	PBM SD	PBM Total	Control Mean	Control SD	Control Total	Weight	Mean Difference IV, Random, 95% CI
**1.5.1 Baseline Marginal Bone**								
Bakry et al, 2020	2.4	0.6	6	2.4	1.3	6	0.4%	0.00 [-1.15, 1.15]
Kinalski et al, 2021	2.05	1.32	23	1.64	1.28	27	1.0%	0.41 [-0.31, 1.13]
Lobato et al, 2019	3.86	1.54	25	4.07	1.96	25	0.5%	-0.21 [-1.19, 0.77]
Makaky et al, 2020	0.25	0.0827	20	0.255	0.0887	20	21.5%	-0.01 [-0.06, 0.05]
**Subtotal (95% CI)**			**74**			**78**	**23.4%**	**-0.00 [-0.06, 0.05]**
Heterogeneity: Tau² = 0.00; Chi² = 1.43, df = 3 (P = 0.70); I² = 0%								
Test for overall effect: Z = 0.13 (P = 0.90)								
**1.5.2 12 weeks**								
Bakry et al, 2020	3	1.1	6	3.1	1.7	6	0.2%	-0.10 [-1.72, 1.52]
Gulati et al, 2020	0.81	0.13	10	0.97	0.09	10	16.9%	-0.16 [-0.26, -0.06]
**Subtotal (95% CI)**			**16**			**16**	**17.1%**	**-0.16 [-0.26, -0.06]**
Heterogeneity: Tau² = 0.00; Chi² = 0.01, df = 1 (P = 0.94); I² = 0%								
Test for overall effect: Z = 3.20 (P = 0.001)								
**1.5.3 6 months**								
Bakry et al, 2020	2.8	1	6	3.1	1.3	6	0.3%	-0.30 [-1.61, 1.01]
Elsyad et al, 2019	0.31	0.1443	20	0.4925	0.3172	20	11.8%	-0.18 [-0.34, -0.03]
Kinalski et al, 2021	1.95	1.16	23	1.28	0.95	27	1.4%	0.67 [0.08, 1.26]
Lobato et al, 2019	2.59	1.56	25	3.21	1.74	25	0.6%	-0.62 [-1.54, 0.30]
**Subtotal (95% CI)**			**74**			**78**	**14.1%**	**-0.04 [-0.57, 0.49]**
Heterogeneity: Tau² = 0.17; Chi² = 8.54, df = 3 (P = 0.04); I² = 65%								
Test for overall effect: Z = 0.15 (P = 0.88)								
**1.5.4 12 months**								
Elsyad et al, 2019	0.49	0.1818	20	0.715	0.3884	20	9.3%	-0.22 [-0.41, -0.04]
Gulati et al, 2020	0.88	0.16	10	1.04	0.13	10	13.9%	-0.16 [-0.29, -0.03]
Makaky et al, 2020	0.265	0.0745	20	0.275	0.0716	20	22.1%	-0.01 [-0.06, 0.04]
**Subtotal (95% CI)**			**50**			**50**	**45.4%**	**-0.11 [-0.25, 0.03]**
Heterogeneity: Tau² = 0.01; Chi² = 8.77, df = 2 (P = 0.01); I² = 77%								
Test for overall effect: Z = 1.58 (P = 0.11)								
**Total (95% CI)**			**214**			**222**	**100.0%**	**-0.09 [-0.16, -0.01]**
Heterogeneity: Tau² = 0.01; Chi² = 28.33, df = 12 (P = 0.005); I² = 58%								
Test for overall effect: Z = 2.35 (P = 0.02)								
Test for subgroup differences: Chi² = 8.56, df = 3 (P = 0.04), I² = 64.9%								

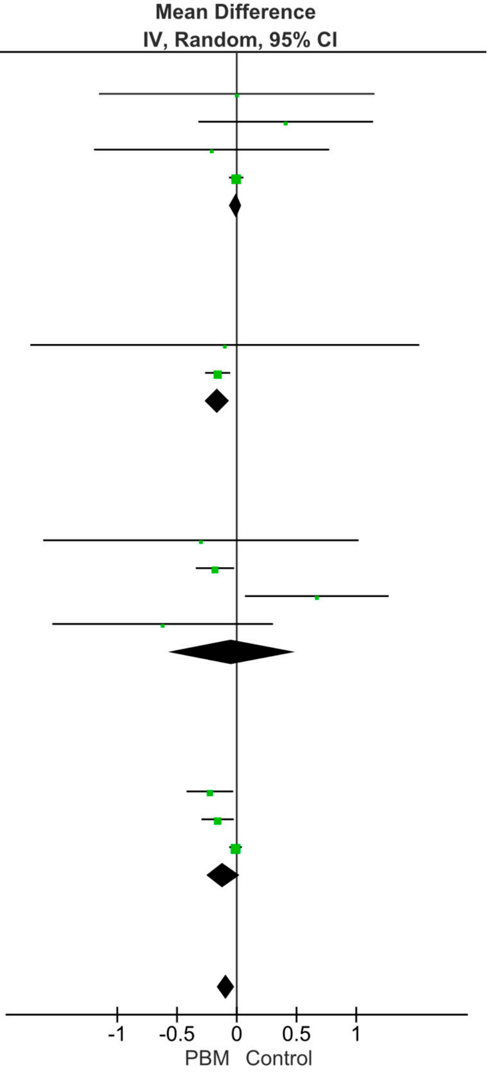

S2F — Postoperative Pain (VAS/NRS)

Supplementary Figure S2F. Forest plot for postoperative pain. Negative mean differences indicate reduced pain with PBM.

Study or Subgroup	PBM Mean	PBM SD	PBM Total	Control Mean	Control SD	Control Total	Weight	Mean Difference IV, Random, 95% CI
**1.6.1 Baseline Postoperative Pain**								
Karaca et al, 2017	3	1.43	25	3	1.43	25	1.0%	0.00 [-0.79, 0.79]
Makaky et al, 2020	3.45	1.099	20	3.5	0.946	20	1.5%	-0.05 [-0.69, 0.59]
Reza et al, 2021	3.14	1.1	21	3.48	1.12	21	1.4%	-0.34 [-1.01, 0.33]
**Subtotal (95% CI)**			**66**			**66**	**3.9%**	**-0.14 [-0.54, 0.26]**
Heterogeneity: Tau² = 0.00; Chi² = 0.54, df = 2 (P = 0.76); I² = 0%								
Test for overall effect: Z = 0.69 (P = 0.49)								
**1.6.2 1 day**								
Collado Murcia et al, 2024	2.07	2.72	31	2.1	2.31	31	0.4%	-0.03 [-1.29, 1.23]
Karaca et al, 2017	4.9	2.03	25	4.9	2.03	25	0.5%	0.00 [-1.13, 1.13]
Makaky et al, 2020	5	1.124	20	5.2	0.834	20	1.7%	-0.20 [-0.81, 0.41]
Makker et al, 2024	3.8	0.45	5	4.6	0.55	5	1.6%	-0.80 [-1.42, -0.18]
Reza et al, 2021	1.9	1	21	2.2	1	21	1.7%	-0.30 [-0.90, 0.30]
Zou et al, 2024	4	3	35	4	3	35	0.3%	0.00 [-1.41, 1.41]
**Subtotal (95% CI)**			**137**			**137**	**6.2%**	**-0.35 [-0.66, -0.03]**
Heterogeneity: Tau² = 0.00; Chi² = 3.12, df = 5 (P = 0.68); I² = 0%								
Test for overall effect: Z = 2.14 (P = 0.03)								
**1.6.3 2 days**								
Collado Murcia et al, 2024	1.97	2.75	31	1.65	1.02	31	0.6%	0.32 [-0.71, 1.35]
Makker et al, 2024	2.4	0.55	5	3.4	0.55	5	1.3%	-1.00 [-1.68, -0.32]
Zou et al, 2024	3	2	35	3	3	35	0.4%	0.00 [-1.19, 1.19]
**Subtotal (95% CI)**			**71**			**71**	**2.4%**	**-0.31 [-1.20, 0.57]**
Heterogeneity: Tau² = 0.37; Chi² = 5.16, df = 2 (P = 0.08); I² = 61%								
Test for overall effect: Z = 0.69 (P = 0.49)								
**1.6.4 3 days**								
Karaca et al, 2017	4	1.72	25	4	1.7	25	0.7%	0.00 [-0.95, 0.95]
Makaky et al, 2020	4.7	1.081	20	4.8	0.894	20	1.7%	-0.10 [-0.71, 0.51]
Makker et al, 2024	1	0.71	5	2	0.71	5	0.8%	-1.00 [-1.88, -0.12]
Reza et al, 2021	0.9	0.8	21	1.2	0.9	21	2.4%	-0.30 [-0.82, 0.22]
Zou et al, 2024	2	1	35	2	2	35	1.1%	0.00 [-0.74, 0.74]
**Subtotal (95% CI)**			**106**			**106**	**6.7%**	**-0.25 [-0.56, 0.05]**
Heterogeneity: Tau² = 0.00; Chi² = 3.76, df = 4 (P = 0.44); I² = 0%								
Test for overall effect: Z = 1.61 (P = 0.11)								
**1.6.5 5 days**								
Karaca et al, 2017	2.6	1.16	25	2.6	1.16	25	1.5%	0.00 [-0.64, 0.64]
Makaky et al, 2020	2.85	0.875	20	2.95	0.999	20	1.8%	-0.10 [-0.68, 0.48]
Zou et al, 2024	0.3	0.1	21	0.6	0.2	21	68.4%	-0.30 [-0.40, -0.20]
**Subtotal (95% CI)**			**66**			**66**	**71.7%**	**-0.29 [-0.38, -0.20]**
Heterogeneity: Tau² = 0.00; Chi² = 1.23, df = 2 (P = 0.54); I² = 0%								
Test for overall effect: Z = 6.06 (P < 0.00001)								
**1.6.6 7 days**								
Collado Murcia et al, 2024	0.4	0.86	31	0.61	1.02	31	2.8%	-0.21 [-0.68, 0.26]
Karaca et al, 2017	1.8	0.85	25	1.8	0.85	25	2.8%	0.00 [-0.47, 0.47]
Makaky et al, 2020	1.45	0.605	20	1.55	0.759	20	3.5%	-0.10 [-0.53, 0.33]
**Subtotal (95% CI)**			**76**			**76**	**9.1%**	**-0.10 [-0.37, 0.16]**
Heterogeneity: Tau² = 0.00; Chi² = 0.38, df = 2 (P = 0.83); I² = 0%								
Test for overall effect: Z = 0.77 (P = 0.44)								
**Total (95% CI)**			**522**			**522**	**100.0%**	**-0.27 [-0.35, -0.19]**
Heterogeneity: Tau² = 0.00; Chi² = 17.24, df = 22 (P = 0.75); I² = 0%								
Test for overall effect: Z = 6.73 (P < 0.00001)								
Test for subgroup differences: Chi² = 2.36, df = 5 (P = 0.80), I² = 0%								

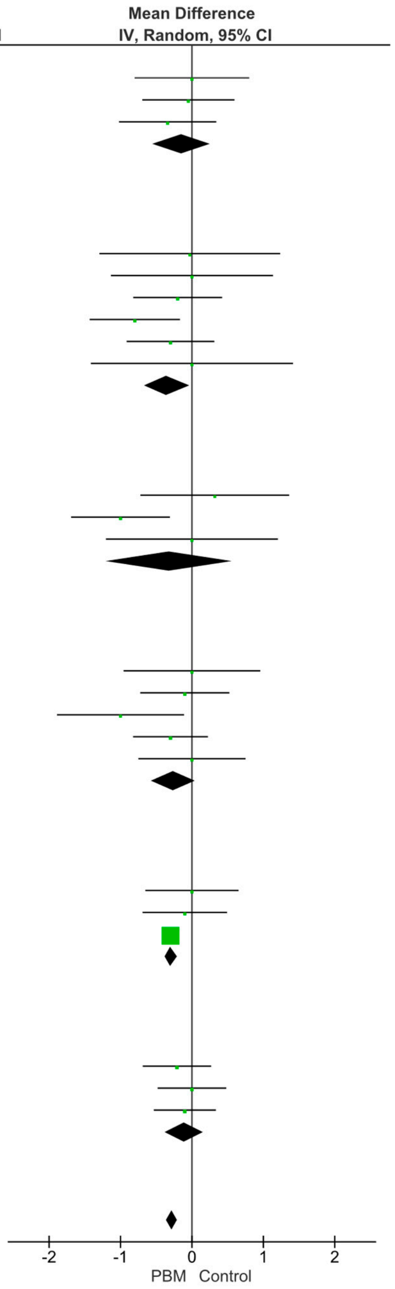

SUPPLEMENTATY FIGURE S3A – FUNNEL PLOT FOR PUBLICATION BIAS
Funnel plots evaluating potential publication bias for each continuous outcome included in the meta-analysis. The vertical line represents the pooled effect estimate, and diagonal pseudo–95% confidence limits indicate expected dispersion. Symmetry suggests low publication bias; asymmetry may indicate selective reporting or small-study effects.

S3A — FUNNEL PLOT FOR ISQ OUTCOMES

Supplementary Figure S3A. Funnel plot for implant stability measured by ISQ.

The plot shows slight asymmetry, with several studies positioned outside the expected triangular region. The Egger line is modestly deviated from vertical, indicating possible small-study effects, though symmetry is acceptable overall. The pattern does not suggest major publication bias and is compatible with heterogeneous early ISQ responses to PBM.

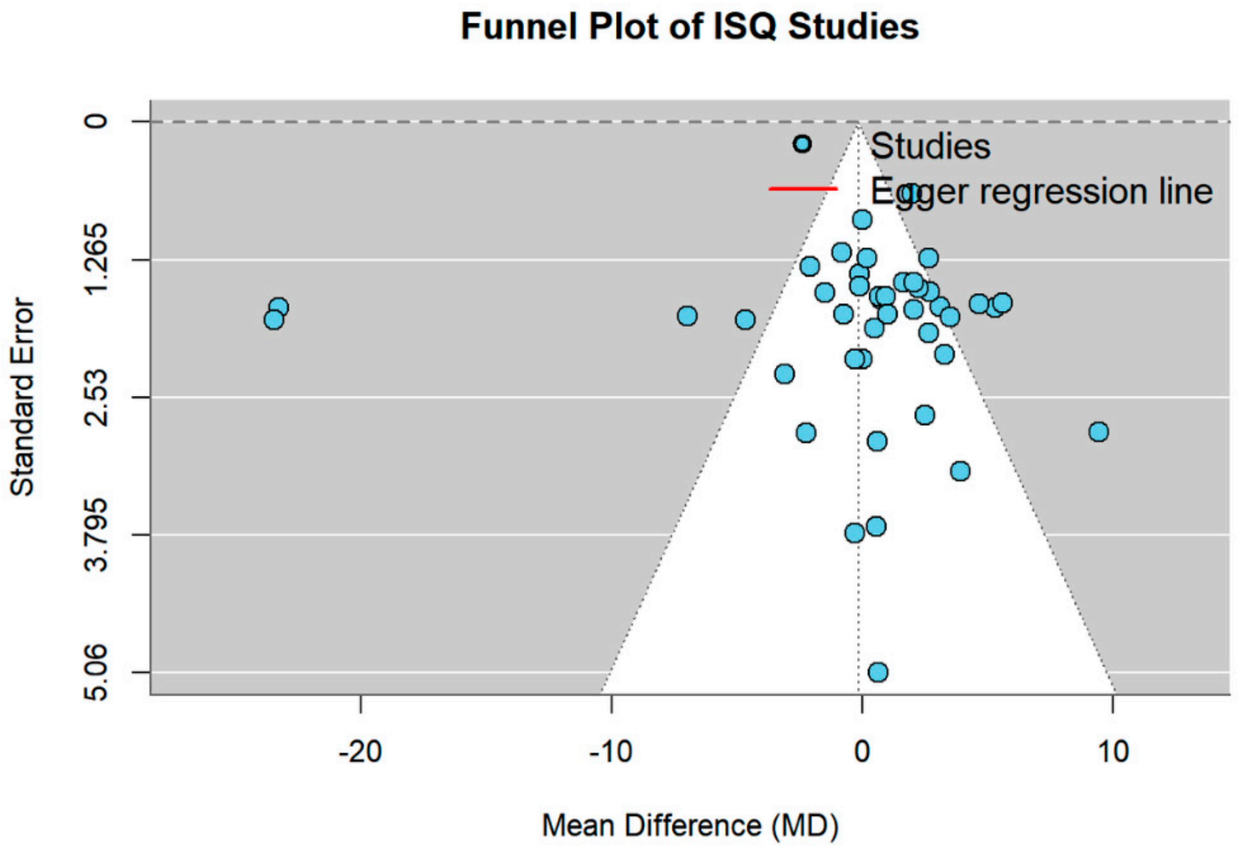

S3B — FUNNEL PLOTS FOR PERIOTEST VALUES

Supplementary Figure S3B. Funnel plot for implant stability measured by Periotest.

A clear asymmetrical pattern is observed, primarily influenced by an extreme outlier with large negative mean difference and higher standard error. Removal of this study would likely restore symmetry. As presented, the plot suggests significant small-study effects, but not systematic publication bias. Outcomes should be interpreted in the context of this distribution.small-study effects, though symmetry is acceptable overall. The pattern does not suggest major publication bias and is compatible with heterogeneous early ISQ responses to PBM.

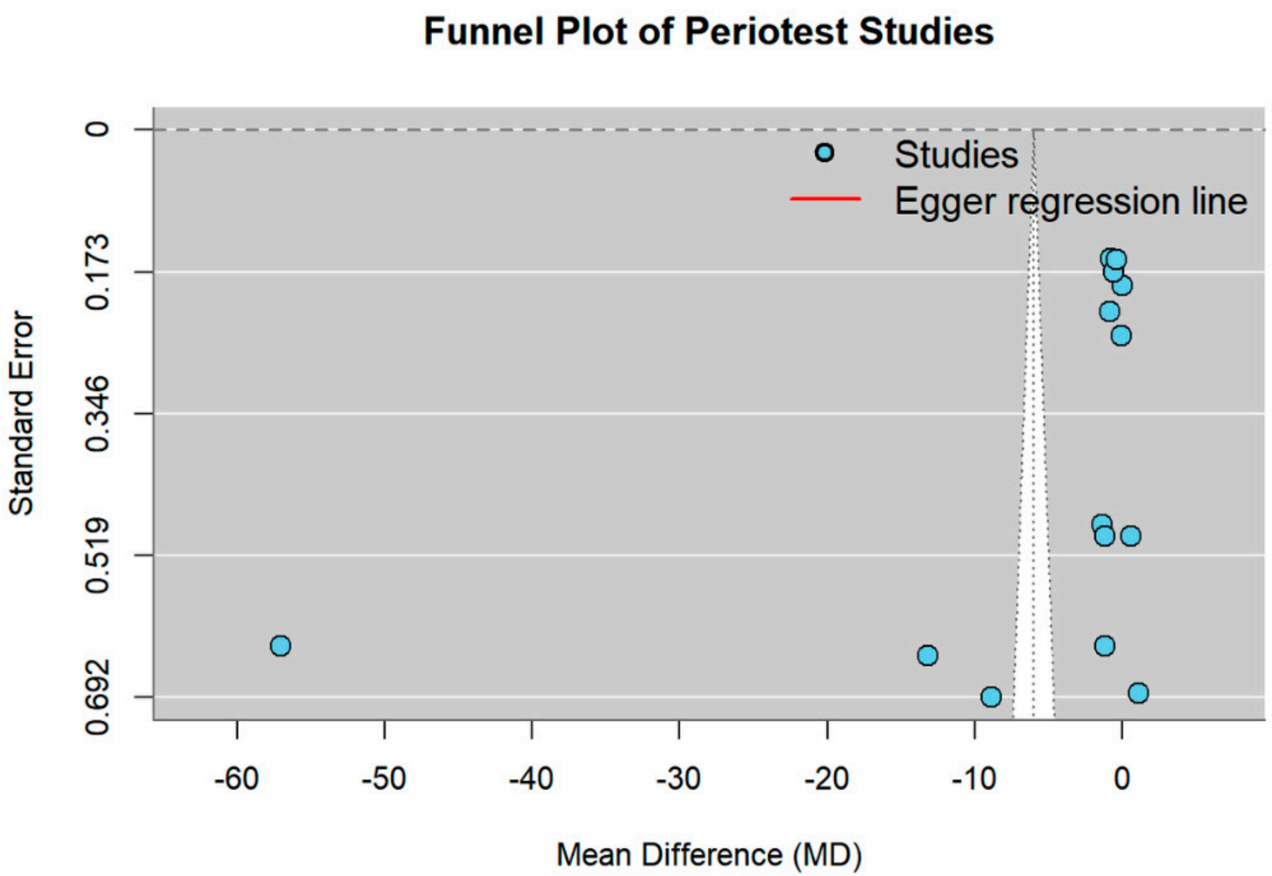

S3C — FUNNEL PLOT FOR BONE DENSITY

Supplementary Figure S3C. Funnel plot for peri-implant bone density outcomes.

The plot demonstrates substantial asymmetry, driven primarily by one outlier study with very large standard error located far from the funnel core. Most studies cluster near the top of the funnel, but the asymmetry indicates a moderate risk of small-study effects or selective reporting. This finding supports caution when interpreting the magnitude of pooled bone density effects.

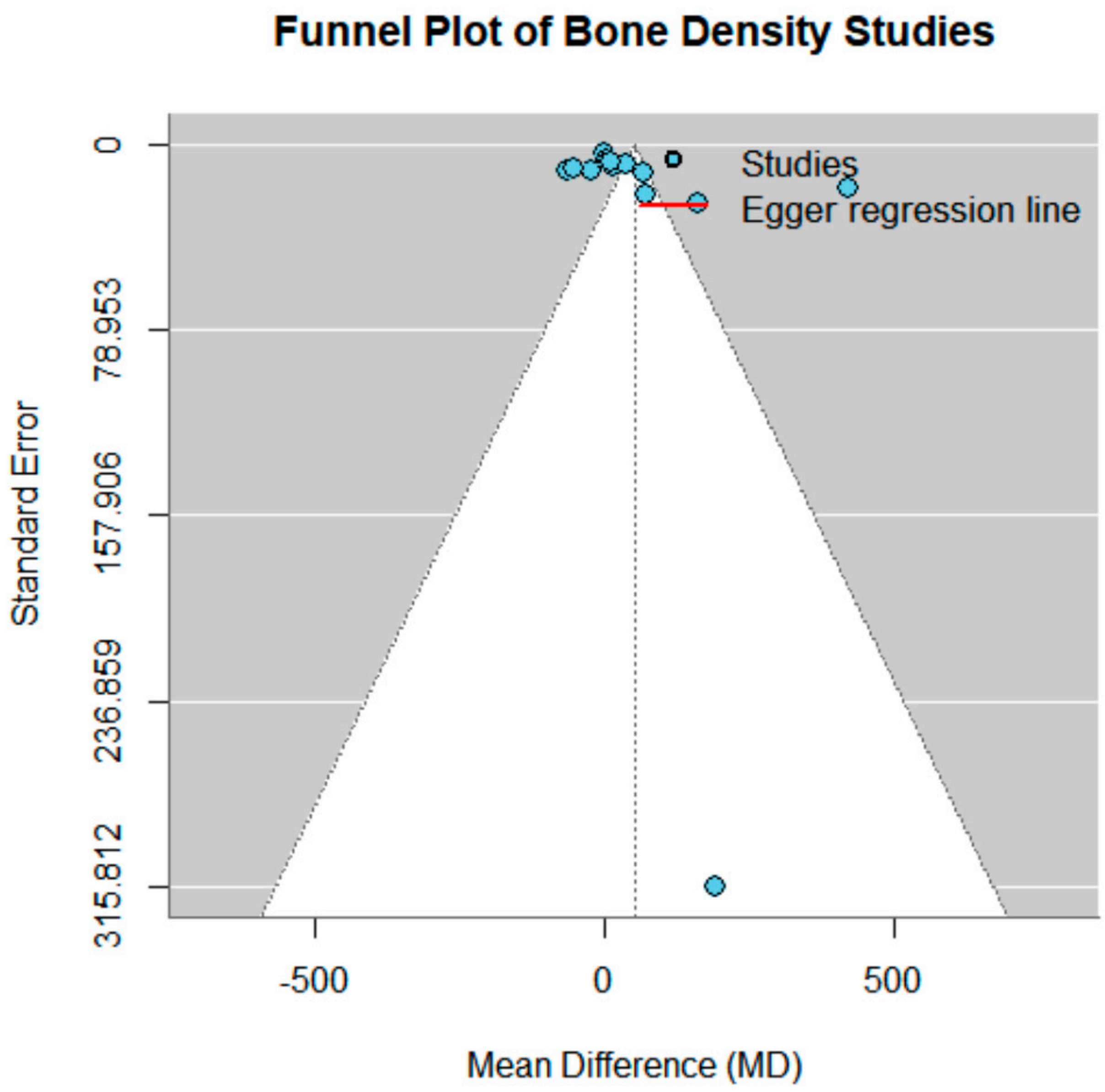

S3D — FUNNEL PLOT FOR MARGINAL BONE LOSS

Supplementary Figure S3D. Funnel plot for marginal bone loss (MBL) outcomes.

The funnel plot reveals notable asymmetry, with multiple smaller studies positioned disproportionately on one side of the mean difference. The Egger regression line reinforces this imbalance, indicating a likely presence of small-study effects, which may contribute to heterogeneity in MBL outcomes. Despite this, no strong directional publication bias is suggested.

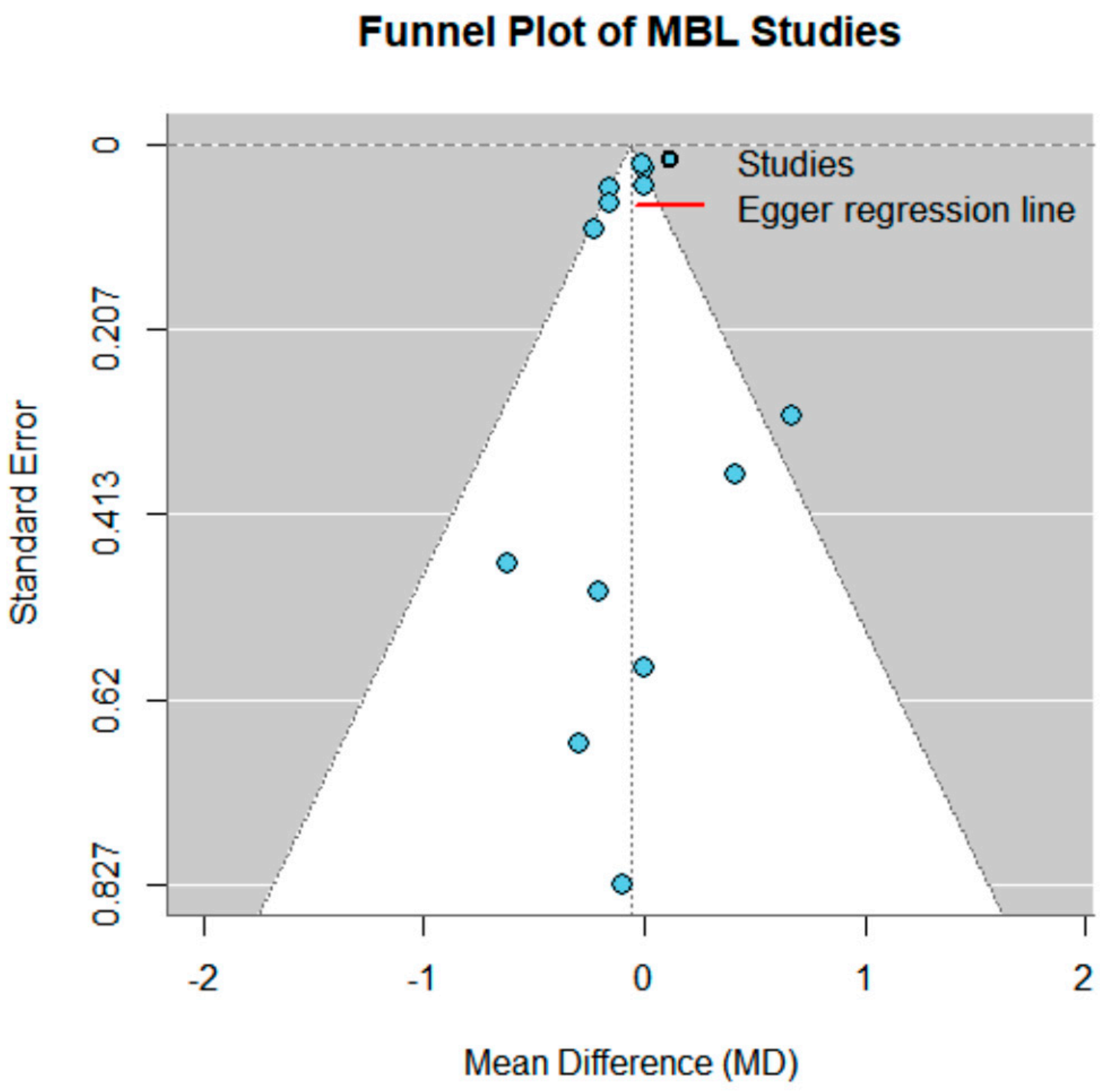

S3E — FUNNEL PLOTS FOR PAIN STUDIES

Supplementary Figure S3E. Funnel plot for postoperative pain outcomes (VAS/NRS).

The distribution of points shows mild asymmetry, with smaller studies dispersed predominantly toward the left side of the funnel. The Egger regression line deviates slightly from the vertical reference, suggesting possible small-study effects, although not a pronounced publication bias. The overall pattern remains largely consistent with PBM reducing postoperative pain.

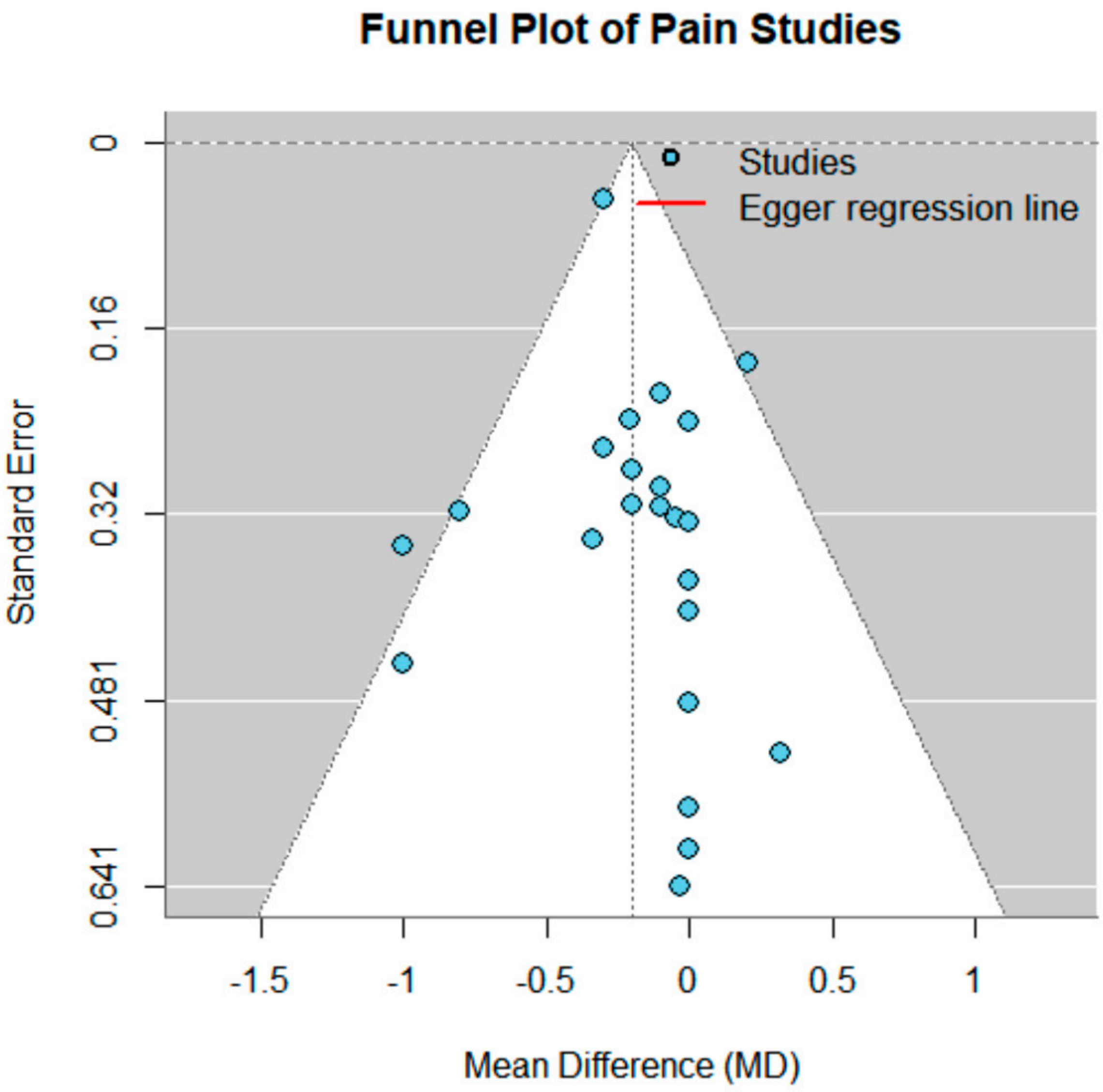

SUPPLEMENTARY TABLE S7 - GRADE EVIDENCE PROFILES

SUPPLEMENTARY S7A - Summary of Findings Table (GRADE Framework) Photobiomodulation in Dental Implant Therapy

Outcome	Nº RCTs	Effect (PBM vs Control)	Risk of Bias	Inconsistency	Indirectness	Imprecision	Publication Bias	Certainty of Evidence
Implant stability (ISQ)	13	MD = 1.26; 95% CI 0.61 to 1.91; favors PBM	Some concerns	Serious (I^2 = 37%)		Serious (wide Cis in subgroups)	Possible	Low
Implant stability (Periotest)	5	MD = −0.55; 95% CI −0.84 to −0.27; favors PBM	Some concerns	Serious (I^2 = 67%)		Not serious	Unlikely	Low
Bone density (3D CBCT / Hounsfield Units)	3	MD = 86.02 HU; 95% CI: -28.48 to 200.52; p = 0.14 (non-significant)	Some concerns	Very serious (I^2 = 98%)	Due to substantial variability in imaging modalities and measurement scales across studies.	Serious (methodological variability)	Possible	Very Low
Bone density (2D radiography / Grayscale)	2	MD = 9.82; 95% CI: 1.08 to 18.56; p = 0.03 (favors PBM)	Some concerns	Very Serious (I^2 = 99%)		Serious (wide CIs)	Possible	Very Low
Marginal bone loss	6	MD = −0.09 mm; 95% CI −0.16 to −0.01; favors PBM	Some concerns	Serious (I^2 = 67%)		Serious (small sample)	Possible	Low
Postoperative pain (VAS/NRS)	6	MD = −0.27; 95% CI −0.35 to −0.19; favors PBM	Some concerns	Not serious (I^2= 0%)		Serious (small effect size)	Unlikely	Moderate

SUPPLEMENTARY S7B - Justifications for Downgrading and Upgrading Decisions (GRADE)

Outcome	Domain downgraded	Justification
ISQ	Risk of bias	Several trials presented "some concerns" in RoB 2 assessment, mainly related to allocation concealment and lack of blinding.
	Inconsistency	Variability across follow-up periods and subgroup analyses reduced confidence in the consistency of the effect, despite only moderate overall heterogeneity (I^2 = 37%).
	Imprecision	Several subgroup estimates presented wide confidence intervals crossing the line of no effect.
Periotest	Risk of bias	Some studies lacked adequate blinding and allocation concealment procedures.
	Inconsistency	Moderate heterogeneity (I^2 = 67%) and variability across follow-up periods reduced certainty, although the overall direction of effect consistently favored PBM.
Bone density	Risk of bias	Serious limitation. Downgraded by one level due to methodological limitations in some of the included trials, specifically regarding a lack of details on allocation concealment and the blinding of outcome assessors (RoB 2).
	Inconsistency	Very serious inconsistency. Downgraded due to high statistical heterogeneity I^2= 99% for the 3D CBCT subgroup and I^2= 67% for the 2D conventional radiography subgroup), combined with substantial clinical and methodological diversity in photobiomodulation (PBM) parameters and follow-up intervals across trials.
	Indirectness	Not serious. No downgrading is warranted for indirectness, as all included randomized controlled trials directly evaluated the target population (adult dental implant patients), the index intervention (photobiomodulation), and the direct clinical outcome of interest (peri-implant bone density). Note: The differences in imaging modalities and measurement scales represent methodological heterogeneity and are addressed under Inconsistency, not Indirectness.

Outcome	Domain downgraded	Justification
MBL	Risk of bias	Variability in imaging acquisition methods and calibration procedures may have influenced outcome assessment.
	Inconsistency	Moderate heterogeneity across studies reduced confidence in the pooled estimate.
	Imprecision	Although statistically significant, the magnitude of effect was very small and of uncertain clinical relevance.
Pain	Risk of bias	Pain outcomes were self-reported, and blinding procedures were not consistently described across studies.
	Imprecision	The magnitude of pain reduction was small despite statistical significance, limiting clinical relevance.

SUPPLEMENTARY TABLE S8A - STANDARDIZED PBM PARAMETERS ACROSS INCLUDED RCTs

PBM Parameter	Values Observed Across Trials	Notes / Interpretation
Device type	GaAlAs, InGaAlP, AlGaInP, LED, Nd:YAG	Diode lasers were predominant.
Wavelength (nm)	630–660 (red); 780–830; 904; 940; 980; 1064	Two main clusters: red (630–660) and infrared (780–830/904–980).
Power output (mW)	20–2000 mW	Higher power generally associated with Nd:YAG or LED clusters.
Irradiance (mW/cm²)	50–354.6 mW/cm²	Influenced by spot size.
Energy per point (J)	0.3–20 J	Wide heterogeneity across protocols.
Fluence (J/cm²)	4–480 J/cm²	Most effective protocols ranged between 10–90 J/cm².
Spot size (cm²)	0.028–1.0 cm²	Determines energy density delivered.
Emission mode	Continuous or pulsed	≈ 90% of trials used continuous mode.
Number of irradiation points	1–20	Multisite irradiation tended to yield better outcomes.
Application timing	Mainly immediate postoperative; some included preoperative or repeated sessions	Single-session protocols less effective.
Total number of sessions	1–15	Serial sessions correlated with stronger effects on pain and stability.

SUPPLEMENTARY TABLE S8B. RECOMMENDED PBM PARAMETERS FOR FUTURE RANDOMIZED CONTROLLED TRIALS

PBM Parameter	Recommended Range / Specification	Rationale / Notes for Future Research
Device type	Diode laser (GaAlAs, InGaAlP) or high-quality LED devices	Stable output, reproducible dosing, good tissue penetration.
Wavelength (nm)	Red: 630–699 nm Near-infrared: 780–980 nm	Optimal absorption by cytochrome c oxidase; best evidence for osteogenic stimulation.
Power output (mW)	20–200 mW	Higher powers increase thermal risk and reduce biological precision.
Irradiance (mW/cm²)	10–200 mW/cm²	Ensures effective photon density without exceeding the inhibitory threshold.
Energy per point (J)	0.5–8 J	Falls within the stimulatory zone of the Arndt–Schulz photobiomodulation curve.
Fluence (J/cm²)	4–50 J/cm²	Supported by preclinical and clinical studies as an effective therapeutic window; avoids supratherapeutic doses.
Spot size (cm²)	0.02–1.0 cm², clearly reported	Essential for accurate dose reporting and reproducibility.
Emission mode	Continuous or pulsed (must be justified and standardized)	Continuous mode is most studied; pulsed mode requires clear parameter reporting.
Number of irradiation points	3–10 points, covering buccal, lingual/palatal, and crestal surfaces	Ensures uniform distribution of light around implant.
Application timing	Combination of intraoperative and early postoperative sessions (first 7–14 days)	Enhances early osteogenic signaling and stability.
Total number of sessions	2–8 sessions	Multi-session protocols result in more consistent outcomes (pain and stability).
Beam geometry reporting	Spot size, angle, distance, and technique must be explicitly described	Required for reproducibility and adherence to laser safety standards.

PBM Parameter	Recommended Range / Specification	Rationale / Notes for Future Research
Treatment justification	Protocol must be grounded in photobiological principles (not arbitrary manufacturer presets)	Ensures translational relevance and methodological rigor.

Note: These recommendations reflect photobiologically supported dosing ranges and do not necessarily represent the full variability observed in existing RCTs (detailed in Supplementary Table S8). Table S8b is intended to guide optimized and standardized PBM protocols for future clinical trials.

SUPPLEMENTARY TABLE S8C. COMPARISON BETWEEN OBSERVED PBM PARAMETERS IN INCLUDED RCTs (S8A) AND RECOMMENDED PARAMETERS FOR FUTURE RCTs (S8B)

PBM Parameter	Values Observed Across RCTs (S8)	Recommended Values for Future Trials (S8b)	Interpretation / Rationale
Device type	GaAlAs, InGaAlP, AlGaInP, LED, Nd:YAG	Diode lasers (GaAlAs, InGaAlP) or medical-grade LED	Trials used many device types → inconsistent energy delivery.
Wavelength (nm)	630–660 (red), 780–830, 904, 940, 980, 1064	630–699 (red) or 780–980 (NIR)	Focus on wavelengths with strongest osteogenic and analgesic evidence.
Power output (mW)	20–2000 mW	20–200 mW	Very high power outputs risk thermal effects and reduce dosing precision.
Irradiance (mW/cm²)	50–354.6 mW/cm²	10–200 mW/cm²	Optimal photon density; avoids overstimulation or inhibition.
Energy per point (J)	0.3–20 J	0.5–8 J	Wide variation in studies; recommended range aligns with biological stimulation curve.
Fluence (J/cm²)	4–480 J/cm²	4–50 J/cm²	Some RCTs used supratherapeutic doses; recommended range prevents inhibitory effects.
Spot size (cm²)	0.028–1.0 cm²	0.02–1.0 cm² (must be reported)	Spot size often missing; essential for reproducibility.
Emission mode	Continuous or pulsed (~90% continuous)	Continuous or standardized pulsed settings	Pulsed mode acceptable but must be justified/reported.
Irradiation points	1–20	3–10	Multisite irradiation preferred for uniform peri-implant stimulation.
Timing of application	Mainly immediate; some repeated sessions	Combined intraoperative + early postoperative (first 7–14 days)	Multi-stage protocols show enhanced stability and reduced pain.
Total sessions	1–15	2–8 sessions	Single session = weaker results; serial dosing = improved outcomes.
Treatment geometry reporting	Incomplete in ~40% of studies	Mandatory reporting (spot size, angle, distance)	Ensures replicability and accurate dosimetry.
Protocol justification	Often lacked photobiological rationale	Must follow photobiological principles	Necessary to reduce heterogeneity and optimize outcomes.

Note: Table S8c summarizes discrepancies between the heterogeneity of PBM parameters found in existing RCTs (Table S8) and the standardized, biologically optimal ranges recommended for future research (Table S8b). This comparison highlights the need for more consistent PBM dosimetry to improve reproducibility and clinical translation.

SUPPLEMENTARY TABLE S9 – RECOMMENDATIONS FOR FUTURE PBM CLINICAL TRIALS IN IMPLANT DENTISTRY

Domain	Recommendation	Rationale
Study design	Use preregistered, double-blind RCTs with proper allocation concealment.	Reduces risk of bias found in current evidence.
Reporting of PBM parameters	Document wavelength, power, fluence, energy per point, spot size, number of points, and total sessions.	Avoids methodological ambiguity and improves reproducibility.
Dosimetry standardization	Target fluence of 10–90 J/cm² adjusted according to tissue depth.	Supported by the most effective trials.
Timing of application	Immediate postoperative + ≥3 additional sessions over 1–2 weeks.	Associated with improved pain and secondary stability.
Anatomical documentation	Report bone density (Lekholm & Zarb), jaw region, cortical thickness.	Reduces heterogeneity across trials.
Outcome measurements	Standardize use of RFA for ISQ, Periotest for mobility, and calibrated CBCT for bone measures.	Ensures comparability and reduces error.
Follow-up duration	Minimum 6 months; ideal 12 months to capture secondary stability and remodeling.	Many trials lacked adequate follow-up.
Pain assessment	Use standardized VAS/NRS collected at identical time points.	Reduces inconsistency in subjective outcomes.
Transparency	Publish full protocols and raw time-point data.	Minimizes publication bias and facilitates future meta-analyses.
